# Seasonality of floral resources in relation to bee activity in agroecosystems

**DOI:** 10.1002/ece3.7260

**Published:** 2021-02-28

**Authors:** Jessica M. Guezen, Jessica R. K. Forrest

**Affiliations:** ^1^ Department of Biology University of Ottawa Ottawa ON Canada

**Keywords:** agricultural landscape, Anthophila, floral volume, pollinators, spatiotemporal scale

## Abstract

The contribution of wild insects to crop pollination is becoming increasingly important as global demand for crops dependent on animal pollination increases. If wild insect populations are to persist in agricultural landscapes, there must be sufficient resources over time and space. The temporal, within‐season component of floral resource availability has rarely been investigated, despite growing recognition of its likely importance for pollinator populations. Here, we examined the visitation rates of common bee genera and the spatiotemporal availability of floral resources in agroecosystems over one season to determine whether local wild bee activity was limited by landscape floral resource abundance, and if so, whether it was limited by the present or past abundance of landscape floral resources. Visitation rates and landscape floral resources were measured in 27 agricultural sites in Ontario and Québec, Canada, across four time periods and three spatial scales. Floral resources were determined based on species‐specific floral volume measurements, which we found to be highly correlated with published measurements of nectar sugar mass and pollen volume. Total floral volume at varying spatial scales predicted visits for commonly observed bee genera. We found *Lasioglossum* and *Halictus* visits were highest in landscapes that provided either a stable or increasing amount of floral resources over the season. *Andrena* visits were highest in landscapes with high floral resources at the start of the season, and *Bombus* visits appeared to be positively related to greater cumulative seasonal abundance of floral resources. These findings together suggest the importance of early‐season floral resources to bees. *Megachile* visits were negatively associated with the present abundance of floral resources, perhaps reflecting pollinator movement or dilution. Our research provides insight into how seasonal fluctuations in floral resources affect bee activity and how life history traits of bee genera influence their responses to food availability within agroecosystems.

## Graphical abstract

If wild bee populations are to persist in agricultural landscapes, there must be sufficient floral resources over time and space. We ask whether the spatial and within‐season temporal availability of floral resources can predict bee activity in agricultural landscapes. We found that visits from sweat bees, mining bees, and bumble bees were best predicted by the amount of floral resources available previously in the season, while visits from leafcutter bees were best predicted by the current availability of floral resources.

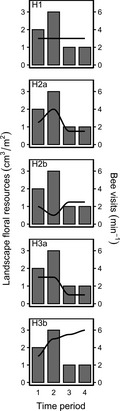

## 1 INTRODUCTION

The abundance and accessibility of floral resources has been identified as the primary factor limiting wild bee populations globally (Roulston & Goodell, 2011). Specifically, if wild bee populations are to persist, there must be sufficient provision of floral resources over both time and space. However, extensive conversion of natural habitat to arable land to support the growing human population has removed many of the naturally occurring floral resources on which wild bee populations rely (Brosi et al., 2008; Kremen et al., 2002; Murray et al., 2009). Even if crops themselves provide floral resources, they do so for only a portion of the growing season, which may be insufficient to support bee populations throughout their activity periods. An abundance of research looking at spatial provisioning of floral resources has generally found that increasing either heterogeneity or abundance of floral resources results in increased population sizes or visitation rates of wild bees (synthesized in Kennedy et al., 2013). However, a few recent studies have found the opposite, with certain floral resource‐providing habitats actually attracting bees away from crop fields (Nicholson et al., 2019), or causing a dilution of pollinators across landscapes when floral resources are less limited (Holzschuh et al., 2016; Kovács‐Hostyánszki et al., 2013).

While the influence of spatial arrangement of floral resources on bees foraging in agricultural landscapes has been well established, the influence of floral resource availability over time has been relatively understudied. Much of the existing research on the latter topic has found that in landscapes providing a consistent source of floral resources over time, wild bees respond positively in terms of their abundance (Mallinger et al., 2016; Mandelik et al., 2012; Martins et al., 2018), density in crops (Kovács‐Hostyánszki et al., 2013), colony growth (Crone & Williams, 2016; Westphal et al., 2009), and sexual reproduction (Rundlöf et al., 2014). However, most studies examining the effect of temporal and spatial arrangement of floral resources on bees focus on responses of abundant, social taxa such as honey bees (Lau et al., 2019) and bumble bees (Timberlake et al., 2019), or examine the responses of broad functional groups of bees, often by grouping solitary bees together (Kovács‐Hostyánszki et al., 2013; Le Féon et al., 2013). An increase in bee population size or density in landscapes with high floral resources can only be observed within one season if bees produce multiple broods per season, or if there is immigration from adjacent landscapes. For wild bee species that have limited flight distances and produce a single brood annually—as is the case for most species in temperate regions—we would expect population sizes to remain stable when floral resource abundance is consistent or increases over a season, and to decrease in response to periods in a season when resources become scarce. Given the differences in brood production, foraging periods, and foraging ranges among bee taxa, fluctuations in floral resources should produce a diversity of responses (Ogilvie & Forrest, 2017). We therefore expect that the spatial and temporal scale of floral resources that most influences bee population size should be specific to the taxonomic group of bees that is examined. Understanding the responses of specific bee taxa to seasonal floral resources in agricultural landscapes is important for development of conservation and management strategies that can both enhance pollination services and preserve bee functional diversity.

**Figure 1:**
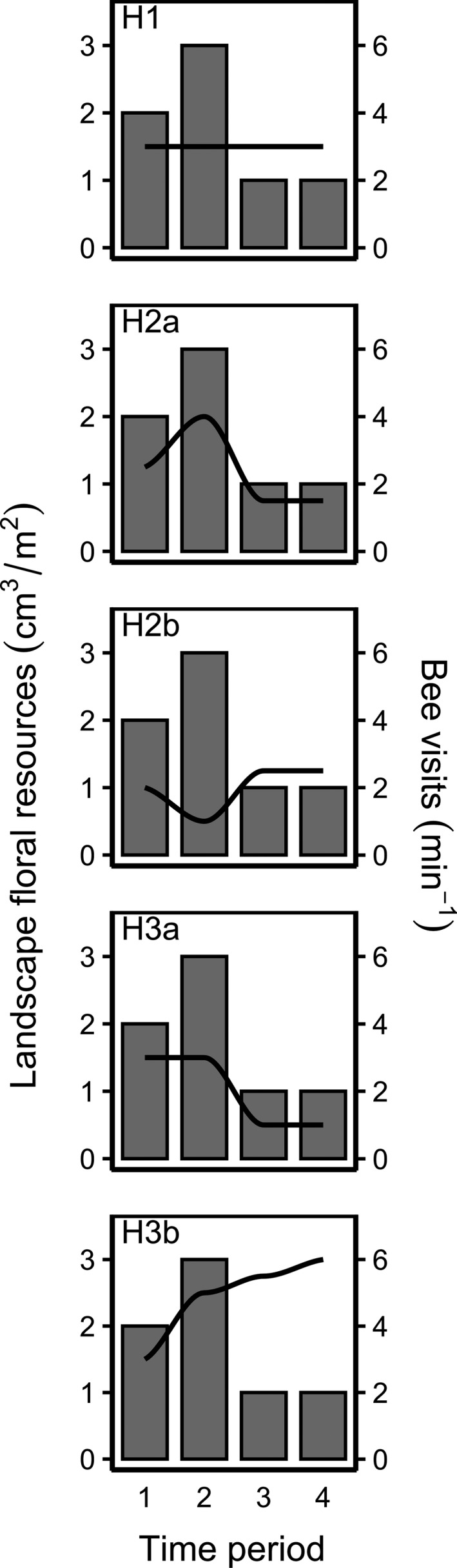
Hypothetical responses of bee visits (in transects) to the amount of floral resources in the surrounding landscape in four time periods over one season, with floral resource abundance represented as gray bars, and bee visits represented as black lines, with hypotheses depicted as follows: H1 = bee visits are only influenced by the present abundance of local floral resources; H2a = bee visits are limited by present floral resource abundance, so that in landscapes with higher floral resource abundance more bee visits are observed; H2b = bee visits are not limited by present floral resource abundance, but are dispersed across landscapes with higher floral resource abundance, in which case fewer bee visits are observed at a local flower patch; H3a = bee visits are influenced by both the abundance of floral resources in the landscape when foraging begins and any decreases in the abundance of floral resources later in the season; and H3b = bee visits are influenced by the cumulative abundance of landscape floral resources from when foraging begins

The primary objective of this study was to examine the relationship between visitation rates of different bee genera and the amount of floral resources in agricultural landscapes over one season, to determine at which within‐year temporal scale and landscape spatial scale the abundance of floral resources predicts local bee abundance. To address this primary objective, we needed to quantify the floral resources available to bees at a landscape scale. Thus, a secondary objective was to find a reliable and practical measurement of floral dimensions that could serve as a proxy for quantities of pollen and nectar sugar. We examined visitation rates of the most common genera of bees and the corresponding amount of floral resources in surrounding agricultural landscapes in four sequential time periods over one season, to assess the relative support for the following hypotheses (presented in Figure 1):

**Bee visits are not influenced by floral resources**. For bee populations that are not limited by the quantity of floral resources (as measured here, using floral dimensions) but are instead limited by factors such as nesting habitat, pesticides, natural enemies, or competition with other bees (resulting in depletion of pollen and nectar), population sizes should not be correlated with the amount of floral resources at the local or landscape scale.

**Bee visits are only influenced by the present abundance of local floral resources**. For bee populations that are not influenced by the amount of floral resources in the broader landscape, we expect that the present abundance of local floral resources (i.e., within the area in which bee visits are measured) will best predict local visitation rates. H0 and H1 represent alternative hypotheses for bees that are not influenced by floral resources in the landscape, driven by the extent to which bees can find and thus respond to local high‐resource patches.

**Bee visits are influenced by the present abundance of landscape floral resources**. For bee populations that are influenced by the availability of floral resources, but that have relatively short foraging periods within a season, we expect that the present abundance of floral resources within the landscape will best predict bee visits, and that either (a) bees are limited by floral resources, so that in landscapes with higher floral resource abundance more bee visits will be observed; or that (b) bees are not limited by floral resources, but instead are “diluted” (dispersed) across landscapes with higher floral resource abundance, in which case fewer bee visits will be observed at a local flower patch.

**Bee visits are influenced by the previous abundance of landscape floral resources**. For bee populations that are influenced by floral resources and have long foraging periods, we expect that (a) for bees producing a single brood per season, both the abundance of floral resources in the landscape early in the season when foraging begins and any decreases in the abundance of floral resources later in the season will best predict bee visits; or (b) for bees producing multiple broods per season, the cumulative abundance of landscape floral resources from when foraging begins will best predict bee visits.

## 2 MATERIALS AND METHODS

### 2.1 Study sites and landscape structure

The study was conducted in 27 farms growing fruit or vegetable crops in Eastern Ontario and the Outaouais region of Québec, Canada (map of sites in Figure S1). Farms planning to grow cucurbit crops were chosen initially for inclusion because we wished to focus on pollinator‐dependent, late‐season crops; however, many farms were not able to grow cucurbits due to drought conditions experienced throughout the region. To maximize independence among farm sites (i.e., to minimize the chance that an individual bee could move between farms), chosen farms were 4–211 km apart. Across all farm sites, 102 locations were sampled for bees and floral resource abundance (as described below), with one to six locations per farm, depending on the number of distinct land patches in which resource‐providing flowers were present, and when permission was given from landowners. Sampling locations within patches of land were selected based on the estimated location of the patch's centre or, if the patch was over 25 m wide, was located at least 10 m from an edge. In three patches wider than 25 m, sampling locations less than 10 m from the edge were used due to a complete absence of flowers in bloom in the centre. The distance between sampling locations within a farm ranged from 3.8 m to 1,040 m. Sites were visited in rotation over four time periods during one season in 2016: the first took place in late spring, between May 20 and June 10 (*n* = 38 sampling locations), the second in early summer, from June 10 to July 4 (*n* = 33), the third in mid‐summer, from July 5 to August 1 (*n* = 37), and the fourth in late summer, from August 1 to September 1 (*n* = 39). If sampling locations contained open flowers during more than one sampling period, the same location was sampled in multiple time periods.

The composition of the landscape within 250, 500, and 750 m radii of each sampling location was quantified to estimate landscape‐scale floral resource abundance. The 250–750 m scale has been found in previous studies to be the range at which non‐*Apis* bees respond to landscape structure (Steffan‐Dewenter et al., 2002), and 500 m was chosen as an intermediate spatial scale. Sampling locations within the same farm site (and with overlapping radii at the 750 m scale) were not treated as independent (see Statistical analysis). Within a 750 m radius around each sampling location, the boundaries between land patches were manually digitized in QGIS version 2.18.7, using both waypoints taken on‐site with a Trimble^®^ Juno *SD* handheld GPS unit (Trimble Navigation Limited), and from Google Earth and Bing Aerial satellite imagery.

Each land patch was then categorized by the type of land‐use (hereafter, “land type”), through ground‐truthing and raster imagery from Agriculture and Agri‐Food Canada's (AAFC) 2016 Annual Crop Inventory. Land types fell into three categories: non‐resource land, resource‐providing land, and unknown land (see Table S1 for detailed descriptions of each land type). Non‐resource land was defined as any area that did not provide floral resources, which included crops with exclusively wind‐pollinated flowers and crops with anecdotal or no evidence of bees collecting resources from flowers. Urban and developed land, which comprised approximately 8.5% of all area surrounding sampling locations, was also included in non‐resource land; although residential gardens may provide floral resources for bees, the amount is inconsistent over time and space (Cane, 2005; Matteson et al., 2008) and appeared in our study to be highly variable across locations. Furthermore, other components of urban and developed land (e.g., pavement, mown lawns) are often devoid of floral resources. Resource‐providing land was defined as land areas that provided floral resources for bees at some point during the season and was categorized into 14 different land types (Table S1). Sampling locations were located only within resource‐providing land, and at least one of each resource‐providing land type was sampled during each time period. Unknown land was comprised of areas where we could not determine the crop grown (2.3% of all area surrounding sampling locations); hedgerow (1.8%); crop land where potentially resource‐providing crops were grown, but floral resources were not measured (0.7%); and soybean (10%), which is of uncertain value as a floral resource for bees. There is some anecdotal evidence for cross‐pollination by honey bees resulting in increased soybean yields (Ahrent & Caviness, 1994; Erickson et al., 1978), and 29 species of wild bees (including eight of the species observed in this study) have been found visiting soybean in Delaware, Wisconsin, and Missouri, USA (Rust et al., 1980). However, many varieties of soybean are cleistogamous, or self‐fertilize before flowers open, and insect pollination of Ontario‐grown varieties is not expected to increase yields (OMAFRA, 2015).

### 2.2 Bee observations

Bee observation methods were adapted from frequently used pollinator surveying designs (Alarcón et al., 2008; Gibson et al., 2011; Memmott, 1999). At each sampling location, a transect was set up to survey bee activity within a 30 m × 4 m area (89 transects); a 30 m × 2 m area was surveyed when only one crop row (< 4 m wide) was present (eight transects); and 25 m × 4 m (one transect) or 24 m × 4m areas (four transects) were surveyed when crop rows were shorter than 30 m. Bee observations occurred over 1 min per 4 m^2^ of transect intervals by slowly walking the length of the transect. The shaded and unshaded temperature, maximum wind speed, and average wind speed were recorded for at least 1 min using a Kestrel^®^ 2000 Pocket Weather^®^ Meter (Nielsen‐Kellerman) held at approximately 1.5 m above ground preceding each observation period. If there was a noticeable change in conditions during the observation period, temperature and wind speed were recorded again at the end of the period and averages were recorded. All bee observations were conducted when shaded temperatures were above 11.9°C (mean ± *SD* = 25.3°C ± 4°C), average wind speeds were below 1.9 m/s, and maximum wind speeds were below 4 m/s.

During observation periods, all occurrences of bees visiting open flowers were recorded by two observers, standing on either side of the transect width, and recording all visits within 2 m each. A visit was counted when a bee was seen contacting sexual organs of an entomophilous flower or was probing a flower for nectar. All visited flowers were identified to genus (9 out of 77 taxa) or species (68 out of 77 taxa), and bees were identified on the wing to genus or species. When identification was not possible on the wing, observations were paused and both observers attempted to catch the bee to take a photograph from inside a glass vial or to collect it as a voucher (79 specimens total). Vouchers were then identified to species or genus and are stored in the Forrest laboratory's collection at the University of Ottawa (Ottawa, ON, Canada). Overall, 82% of bees were identified to species, 17% to genus, 0.1% to family, and 1% as Anthophila. The full list of bee taxa can be found in Table S2.

### 2.3 Floral resources

Floral density was recorded at each sampling location, using three quadrats of 1.5 m × 1.5 m. Quadrats were placed in random locations within the same transect used for bee observations, immediately following the observation period. If no open flowers were present in all three quadrat locations, an additional location was randomly selected and the mean count across the four quadrats was recorded. Within a quadrat, the number of open flowers was counted for each nongraminoid species encountered; for species with many‐flowered inflorescences, five individuals were haphazardly selected, and the number of flowers was counted on a randomly selected inflorescence. The mean number of flowers per inflorescence for many‐flowered species was then multiplied by the number of inflorescences in a quadrat to obtain the number of flowers per quadrat. In members of the Asteraceae family, capitula were treated as single flowers (see Table S3 for descriptions of floral units used for counts of each species). For 29 out of 96 species encountered, the number of flowers per inflorescence was obtained from either literature sources or digital images of herbarium specimens because of the large number of flowers encountered in the field or because (in a few cases) the species was inadvertently overlooked in the field (see Table S3 for literature values for each species).

**Figure 2:**
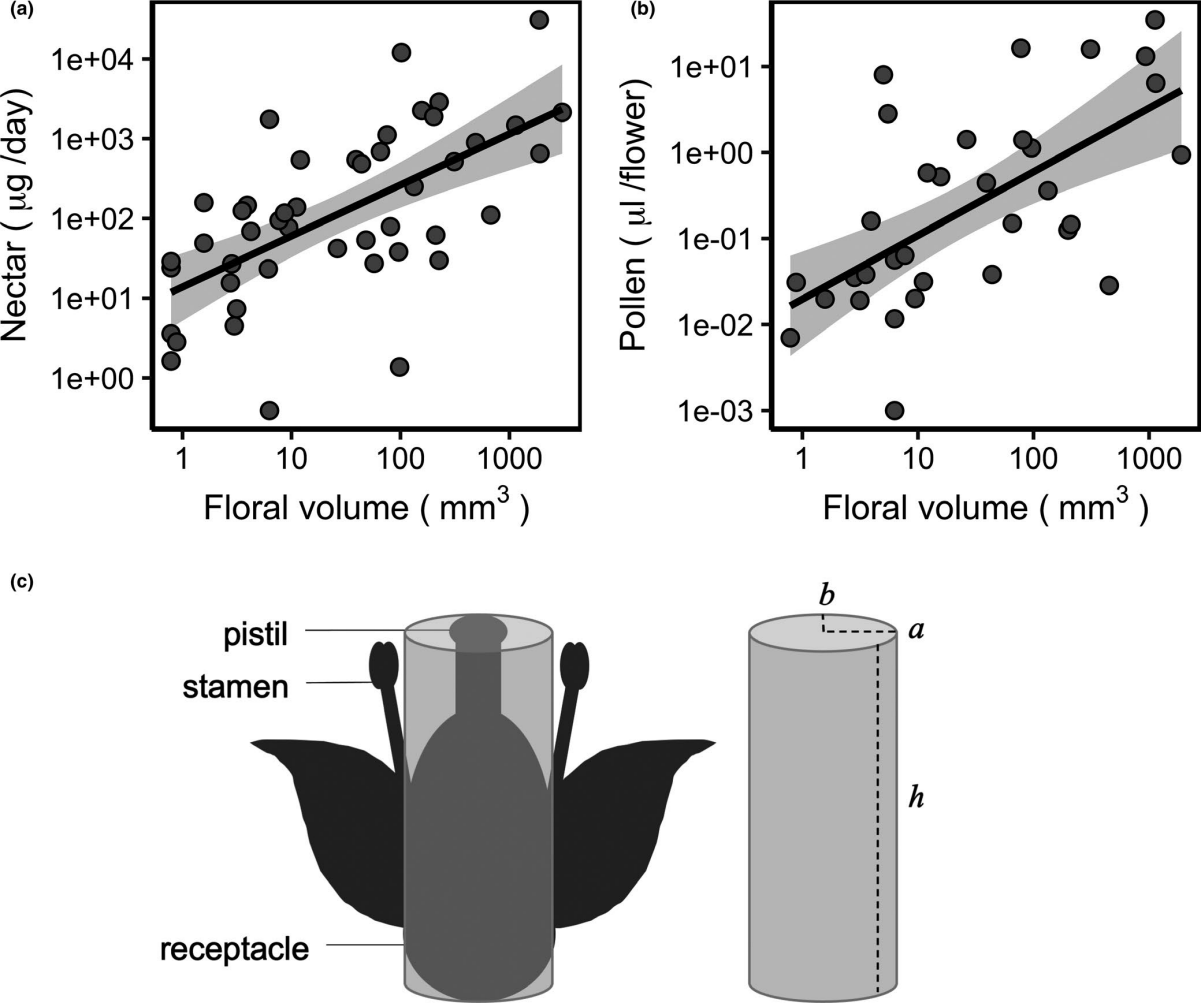
Correlations between floral volume and (a) daily nectar sugar mass in 46 flowering species, and (b) pollen volume in 33 flowering species; with (c) a diagram of floral volume measurements using the volume of an elliptic cylinder (V=πabh). Black lines in (a) and (b) represent the ordinary least squares regression fit of nectar mass or pollen volume by floral volume; shaded areas are 95% confidence intervals. Note logarithmic axes

To estimate the amount of floral resources (nectar and pollen) provided by a species, floral dimensions were measured on five haphazardly selected individuals of each species. The length and width of the receptacle (or capitulum in Asteraceae species) were measured at right angles to each other, as well as the height from the receptacle to the end of the longest sexual organ (stamen or pistil); in species with sexual organs completely hidden within a corolla, height was measured from the receptacle to the end of the corolla. Measurements were made using calipers and were rounded to the nearest 1 mm. Thirty‐one of 96 species were not measured in the field, and floral measurements were instead obtained from literature sources or digital images of herbarium specimens (see Table S3 for measurements and literature sources for each species). Floral measurements were used to calculate both the surface area (*A*) of flowers:

(1) A=πab

and the volume (*V*) of flowers:

(2) V=πabh

where *a* is the semi‐major axis, or half the length or width (whichever was longest) of a flower's receptacle or capitulum, *b* is the semi‐minor axis, or half the length or width (whichever was shortest) of a flower's receptacle or capitulum, and *h* is the height of a flower or inflorescence (Figure 2c and Table S3).

To determine which measurement of floral dimensions was the best proxy for floral resource amount, literature searches for daily nectar sugar mass (µg/day) and pollen volume (in µl/flower) were conducted for all flowering species encountered; these measurements have been previously used to assess floral resources available to pollinators (Baude et al., 2016; Hicks et al., 2016). Literature sources that provided counts of pollen grains per flower and volumes of individual pollen grains were used to calculate an estimate of pollen volume per flower for species for which we could not find measurements of total pollen volume. Nectar sugar mass was obtained for 46 species and pollen volume for 33 species of the 96 encountered (see Tables S4–S5 for full species lists). Pearson correlations between nectar sugar mass or pollen volume and the length, width, height, surface area, and volume measurements of each species (all variables log‐transformed to approximate normal distributions) were used to determine which floral dimension could best estimate the amount of floral resources. In addition, to determine whether the source of floral volume measurements (literature, in‐field measurements, or combination of both; see Table S3) influenced the relationship between floral volume and either nectar or pollen, we ran ANCOVAs on daily nectar sugar mass and pollen volume as functions of floral volume, measurement source, and their interaction. The interaction was nonsignificant for both nectar (*F*_2,40_ = 0.82, *p* = .45) and pollen (*F*_2,28_ = 0.18, *p* = .83), indicating that it was reasonable to combine data sources.

For all bee genera other than *Peponapis*, the abundance of floral resources in the landscape surrounding each sampling location was calculated by determining the mean floral resource value per flower of each species and multiplying this value by the count of each flower in a quadrat. *Peponapis* collect pollen exclusively from squash (*Cucurbita* spp.; Hurd et al., 1974), and we only observed *Peponapis* visiting squash and cucumber (*Cucumis sativus*) flowers (both Cucurbitaceae). Therefore, in models of *Peponapis* visits, the abundance of floral resources in the landscape surrounding each sampling location was calculated from the mean floral resource value per squash or cucumber flower. While other bee genera such as *Andrena* likely included some oligolectic (pollen‐specialist) species, we included all rewarding plant taxa in calculations of floral resources for genera other than *Peponapis*, as oligolectic species would make up a much smaller proportion of the total than in *Peponapis*. Furthermore, collectively, all oligolectic species within other genera would likely be specialized on pollen from multiple taxonomic groups, rather than a single family as in *Peponapis*. The mean abundance of floral resources per 1 m^2^ was then calculated across quadrats for each transect, and the median of the transect‐level values was calculated for each land type during each time period. This number was then multiplied by the total area of each land type within 250, 500, and 750 m around a sampling location to obtain an estimate of the total floral resources at a given spatial scale during a given time period.

### 2.4 Statistical analysis

All statistical analyses were performed in R version 3.6.1 (R Core Team, 2019). Analyses of bee visitation rate per transect were conducted on individual bee genera that were present in at least four out of 27 sites during a given time period (Table 1) and on the entire bee community. (Note that data from all 27 sites were included in each analysis; i.e., visitation rates of zero for a given taxon at a given site were considered meaningful and were included in analysis.) To check for multicollinearity, we calculated Pearson correlation coefficients as well as VIF values using the performance package (Lüdecke et al., 2019). Pearson correlation coefficients were between –0.37 and 0.23 (Table S6) and all VIF values were ≤4.89, indicating acceptably low correlation. Spatial autocorrelation among sites in the number of visits by each genus in a given time period was assessed using Moran's I (Paradis et al., 2004). Visits from *Apis mellifera* were spatially autocorrelated across all time periods (*p* = .02, Moran's I observed – expected = 0.14), likely due to the presence of hives on certain farms, so were not analyzed.

**Table 1:** Number of visits and presence across sites (*n* = 27) for all bee genera during each time period (T1 = late spring, T2 = early summer, T3 = mid‐summer, T4 = late summer) and over the whole season (“Total”)

Bee genera	Visits					Sites present				
	T1	T2	T3	T4	Total	T1	T2	T3	T4	Total
*Andrena*	154	117	229	16	516	9	13	10	4	23
*Apis*	794	742	433	486	2,455	11	11	16	14	22
*Augochlora*	0	9	0	0	9	0	3	0	0	3
*Augochlorella*	43	25	8	8	84	8	6	3	3	14
*Bombus*	263	406	606	940	2,215	11	17	17	21	26
*Ceratina*	40	26	34	12	112	3	5	2	3	10
*Colletes*	0	7	2	2	11	0	2	1	1	3
*Halictus*	111	249	159	18	537	10	20	6	4	22
*Heriades*	0	0	3	0	3	0	0	1	0	1
*Hoplitis*	0	12	9	0	21	0	2	2	0	4
*Hylaeus*	2	28	0	0	30	1	2	0	0	3
*Lasioglossum*	200	225	166	31	622	18	17	16	12	26
*Megachile*	0	47	52	19	118	0	8	5	3	12
*Melissodes*	0	2	0	25	27	0	1	0	3	4
*Osmia*	26	10	4	0	40	4	1	1	0	5
*Peponapis*	0	2	482	1,035	1,519	0	1	12	16	20
*Perdita*	0	0	0	12	12	0	0	0	1	1
*Sphecodes*	0	1	0	3	4	0	1	0	1	1

To model the number of bee visits observed for each taxonomic group, generalized linear mixed models were run with a zero‐inflated negative binomial distribution and log link function, using the glmmTMB package (Brooks et al., 2017). This distribution is typically used with zero‐inflated count data, and a high probability of zero‐inflation was confirmed for each taxonomic group using the performance package (Lüdecke et al., 2019). All models included a log offset to account for varying lengths of observation time based on transect sizes, and the crossed random effects of time period and site to account for the dependency among observations within time periods and sites. The full models for each hypothesis run are presented below in mathematical syntax, followed by R syntax; all iterations of models run are presented in R syntax in Table S7. The full model for the null hypothesis (H0) was of the form.

(3) $$\begin{array}{cc} {\text{Visits}}_{\mathrm{ijk}} & ={\text{AllOtherVisits}}_{\mathrm{ijk}}+\log\left({\text{ObservationTime}}_{\mathrm{ijk}}\right)+{\text{Site}}_{j}+{\text{TimePeriod}}_{k}\\ {\text{Site}}_{j} & ∼N\left(0,\sigma_{\text{Site}}^{2}\right)\\ {\text{TimePeriod}}_{k} & ∼N\left(0,\sigma_{\text{TimePeriod}}^{2}\right)\\ R:\text{Visits} & ∼\text{AllOtherVisits}+\text{offset}(\log(\text{ObservationTime})\text{)}+(1|\text{Site})+(1|\text{TimePeriod}) \end{array}$$

where Visits*_ijk_* is the number of bee visits of a given taxonomic group observed in the *i*th transect in site *j* during time period *k*, AllOtherVisits*_ijk_* is the number of visits observed in the *i*th transect in site *j* during time period *k* from all bees other than the taxonomic group represented in Visits*_ijk_*, ObservationTime*_ijk_* is the length of the observation time in the *i*th transect in site *j* during time period *k*, and Site*_j_* and TimePeriod*_k_* are random intercepts, which are assumed to be normally distributed with mean 0 and variances $\sigma_{\text{Site}}^{2}$ and $\sigma_{\text{TimePeriod}}^{2}$, respectively. The AllOtherVisits*_ijk_* term was included to allow for possible competitive relationships among bees. A reduced version of the null model was also run excluding AllOtherVisits*_ijk_* (Table S7).

All of the following models included the same random intercept and offset terms but differed from Equation (3) in the fixed effects included. The full model for H1 was of the form.

(4) $$\begin{array}{cc} {\text{Visits}}_{\mathrm{ijk}} & ={\text{TransectFR}}_{\mathrm{ijk}}+{\text{AllOtherVisits}}_{\mathrm{ijk}}+\log\left({\text{ObservationTime}}_{\mathrm{ijk}}\right)+{\text{Site}}_{j}+{\text{TimePeriod}}_{k}\\ {\text{Site}}_{j} & ∼N\left(0,\sigma_{\text{Site}}^{2}\right)\\ {\text{TimePeriod}}_{k} & ∼N\left(0,\sigma_{\text{Timeperiod}}^{2}\right)\\ R:\text{Visits} & ∼\text{TransectFR}+\text{AllOtherVisits}+\text{offset}(\log(\text{ObservationTime}))+(1|\text{Site})+(1|\text{TimePeriod}) \end{array}$$

where TransectFR*_ijk_* is the floral resource volume (cube‐root transformed) within the *i*th transect in site *j* during time period *k*; that is, this model includes a term for local but not landscape‐level floral resources. A reduced version of the full H1 model was also run excluding the fixed effect AllOtherVisits*_ijk_* (Table S7). The full model for H2a and H2b, which also includes landscape‐level floral resources, was of the form

(5) $$\begin{array}{cc} {\text{Visits}}_{\mathrm{ijk}} & ={\text{PresentFR}}_{\mathrm{ijk}}+{\text{TransectFR}}_{\mathrm{ijk}}+{\text{AllOtherVisits}}_{\mathrm{ijk}}+\log\left({\text{ObservationTime}}_{\mathrm{ijk}}\right)+{\text{Site}}_{j}+{\text{TimePeriod}}_{k}\\ {\text{Site}}_{j} & ∼N(0,\sigma_{\text{Site}}^{2})\\ {\text{TimePeriod}}_{k} & ∼N(0,\sigma_{\text{TimePeriod}}^{2})\\ R:\text{Visits} & ∼\text{PresentFR}+\text{TransectFR}+\text{AllOtherVisits}+\text{offset}(\log(\text{ObservationTime}))+(1|\text{Site})+(1|\text{TimePeriod}) \end{array}$$

where PresentFR*_ijk_* is the present landscape floral resource volume (cube‐root transformed) within either a 250, 500, or 750 m radius (each spatial scale was run in separate models) surrounding the *i*th transect in site *j* during the current time period *k*. Reduced versions of the full H2 model were also run excluding the fixed effects AllOtherVisits*_ijk_* and/or TransectFR*_ijk_* (Table S7). The full model for H3a was of the form

(6) $$\begin{array}{cc} {\text{Visits}}_{\mathrm{ijk}} & ={\text{FR}}_{ij(1)}+\Delta {\text{FR}}_{\mathrm{ijk}}+{\text{TransectFR}}_{\mathrm{ijk}}+{\text{AllOtherVisits}}_{\mathrm{ijk}}+\text{log}\left({\text{ObservationTime}}_{\mathrm{ijk}}\right)+{\text{Site}}_{j}+{\text{TimePeriod}}_{k}\\ \Delta {\text{FR}}_{\mathrm{ijk}} & =\sum_{k=1}^{k}\left({\text{FR}}_{\mathrm{ijk}}\geq {\text{FR}}_{ij(k‐1)}\to 0\right)\wedge \left({\text{FR}}_{\mathrm{ijk}}<{\text{FR}}_{ij(k‐1)}\to {\text{FR}}_{\mathrm{ijk}}‐{\text{FR}}_{ij(k‐1)}\right)\\ {\text{Site}}_{j} & ∼N(0,\sigma_{\text{Site}}^{2})\\ {\text{TimePeriod}}_{k} & ∼N(0,\sigma_{\text{TimePeriod}}^{2})\\ R:\text{Visits} & ∼\text{FirstTimePeriodFR}+\text{ChangeInFR}+\text{TransectFR}+\text{AllOtherVisits}+\text{offset}(\text{log}(\text{ObservationTime}))+(1|\text{Site})+(1|\text{TimePeriod}) \end{array}$$

where FR*_ij_*_(1)_ (R: FirstTimePeriodFR) is the landscape floral resource volume (cube‐root transformed) within either a 250, 500, or 750 m radius (each spatial scale was run in separate models) surrounding the *i*th transect in site *j* during the first time period that bees were observed visiting flowers; ΔFR*_ijk_* (R: ChangeInFR) is the negative change (positive changes were equivalent to no change, i.e., equaling zero) in the landscape floral resource volume (cube‐root transformed) measured within the same radius as FR*_ij_*_(1)_. ΔFR*_ijk_* was calculated by determining whether landscape floral resource volume from time period *k* was greater than or equal to the volume measured in the previous time period (FR*_ijk_* ≥ FR*_ij_*_(_ *_k_*_–1)_ → 0), or if it was less than the volume measured in the previous time period (FR*_ijk_* < FR*_ij_*_(_ *_k_*_–1)_ → FR*_ijk_* – FR*_ij_*_(_ *_k_*_–1)_). The cumulative ΔFR*_ijk_* was then calculated by summing across all time periods since bees were first observed visiting flowers (*k* = 1). Reduced versions of the full H3a model were run excluding AllOtherVisits*_ijk_* and/or TransectFR*_ijk_* (Table S7). Models of *Peponapis* visits did not include the ΔFR*_ijk_* term because across all sites and time periods it was equal to zero (i.e., only positive or no changes in the floral resource volume were observed from mid‐ to late summer). Models for H3a were only run for bee genera that could produce a single brood per season (*Andrena*, *Halictus*, *Lasioglossum, Megachile*, and *Peponapis*). Finally, the full model for H3b was of the form

(7) $$\begin{array}{cc} {\text{Visits}}_{\mathrm{ijk}} & =\sum_{k=1}^{k}\left({\text{FR}}_{\mathrm{ijk}}\right)+{\text{TransectFR}}_{\mathrm{ijk}}+{\text{AllOtherVisits}}_{\mathrm{ijk}}+\log\left({\text{ObservationTime}}_{\mathrm{ijk}}\right)+{\text{Site}}_{j}+{\text{TimePeriod}}_{k}\;\\ {\text{Site}}_{j} & ∼N(0,\sigma_{\text{Site}}^{2})\\ {\text{TimePeriod}}_{k} & ∼N(0,\sigma_{\text{TimePeriod}}^{2})\\ R:\text{Visits} & ∼\text{CumulativeFR}+\text{TransectFR}+\text{AllOtherVisits}+\text{offset}(\log(\text{ObservationTime})\text{)}+(1|\text{Site})+(1|\text{TimePeriod}) \end{array}$$

where FR*_ijk_* is the landscape floral resource volume (cube‐root transformed) within either a 250, 500, or 750 m radius surrounding the *i*th transect in site *j* during time period *k*, from which the cumulative FR*_ijk_* (R: CumulativeFR) was calculated by summing across all time periods since bees first were observed visiting flowers (including the present time period). Reduced versions of the full H3b model were run excluding AllOtherVisits*_ijk_* and/or TransectFR*_ijk_* (Table S7). Models for H3b were only run for bee genera that might produce multiple broods per season (*Bombus*, *Halictus*, *Lasioglossum*; Packer et al., 2007).

Model selection with the MuMIn package (Bartoń, 2019) was used to determine which hypothesis best predicted bee visits based on AICc values. Model selection was run first to determine the best model (ΔAICc = 0) for a given hypothesis (H0, H1, H2, H3a, or H3b) at a given spatial scale (250, 500, or 750 m for H2, H3a, and H3b). Models were then compared across all hypotheses, to determine which hypothesis was most predictive of bee visits. Any model not reaching convergence or showing significant problems based on residual diagnostic plots (DHARMa package; Hartig, 2020) was excluded from analysis. Model selection was run in two iterations, with unknown land area assigned either the median floral resource value calculated from all land types within a specific radius during a given time period (“median models”), or the minimum floral resource value calculated from all land types within a specific radius during a given time period, which was always zero (“minimum models”). Areas with soybean were only assigned the median value in the early summer and mid‐summer time periods, because soybeans in Ontario were at nonreproductive stages on June 14 in 2016 (OMAFRA, 2016a), and by July 27, most soybeans were in early pod stage (OMAFRA, 2016b); therefore, flowering most likely coincided with only early summer and mid‐summer (between June 10 and August 1). These two iterations were run to test the sensitivity of our conclusions to the presence of unknown areas. The hypothesis with the best support was chosen based on the lowest ΔAICc value when summed across both median and minimum models. For the best supported hypothesis, estimates of coefficients were calculated by averaging across all median or minimum models for a given hypothesis with ΔAICc < 7, using the MuMIn package (Bartoń, 2019).

## 3 RESULTS

### 3.1 Floral resources

Floral volume was the best predictor of both daily nectar sugar mass (µg/day; *r* = .62, *p* = 3.6 × 10^–6^, *n* = 46, Figure 2a) and pollen volume (µl/flower; *r* = .63, *p* = 6.0 × 10^–5^, *n* = 33, Figure 2b), and therefore was used to represent floral resources in all subsequent analyses. Flower length, width, height, and surface area were also significantly correlated with nectar and pollen volume, but to a lesser degree (*r* ≤ .59). Floral measurements, pollen volumes, and nectar sugar mass for individual species can be found in Table S3–S5.

**Figure 3:**
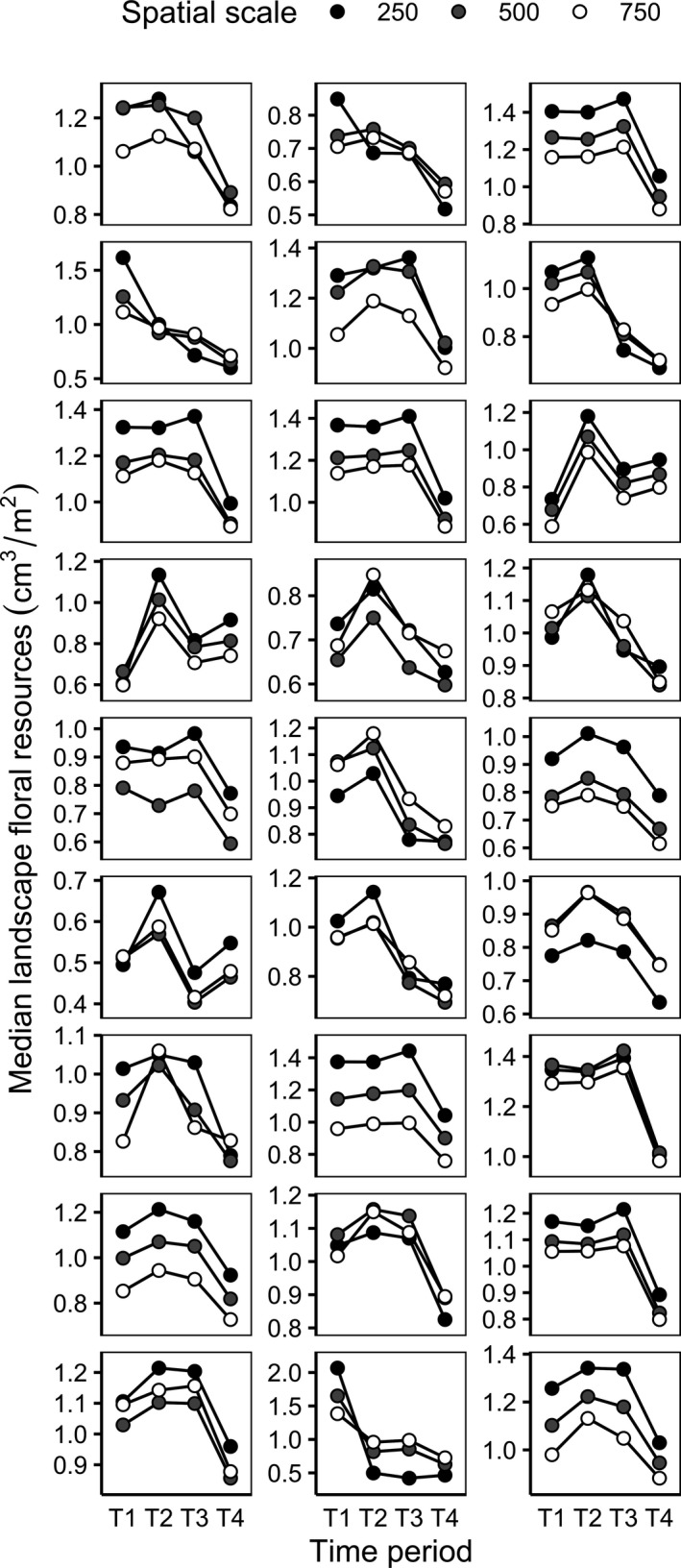
Median volumes of floral resources per square meter within each landscape spatial scale (250 m, 500 m, and 750 m radii around sampling locations) across all time periods (T1 = late spring, T2 = early summer, T3 = mid‐summer, T4 = late summer) in one season, with panels showing trends within each site (*n* = 27). Floral resources were calculated with any unknown areas assigned the median floral resource volume from all resource‐providing land types in a given spatial scale and time period

Across many of the landscapes sampled in this study, there was a high correlation in floral resources between spatial scales within a given time period, particularly between the 500 m and 750 m spatial scales (Figure 3). In most landscapes, the lowest floral resource abundance occurred in late summer, and the highest floral resource abundance generally occurred in either early summer or mid‐summer, with a few landscapes having the highest floral resource abundance of the season in late spring (Figure 3).

### 3.2 Most common bee genera

Over the season, 8,422 bee visits were observed across all sites, with 1,647 visits observed in late spring, 1,946 in early summer, 2,211 in mid‐summer, and 2,618 in late summer. Bees in the genera *Andrena* (mining bees), *Apis* (*A. mellifera*; Western honey bee), *Bombus* (bumble bees), *Halictus* (furrow bees), and *Lasioglossum* (sweat bees) were observed visiting flowers in at least four of the 27 sites surveyed during all time periods. Bees in the genus *Augochlorella* (*A. aurata*; golden green sweat bee) were observed only during late spring and early summer in at least four sites, while *Megachile* (leafcutter bees) were observed in early summer and mid‐summer, and *Peponapis* (*P. pruinosa*; hoary squash bee) in mid‐summer and late summer. All other bee genera found in at least four sites were observed during only one time period (Table 1).

### 3.3 H0: Bee visits are not influenced by floral resources

Across all taxa examined, bee visits did not appear to be strongly related to the number of other bee visits in a transect (Table 2). The null model for *Peponapis* described the number of visits better than models including floral resources when unknown areas were assigned the median volume of floral resources (Table 3); however, this null model did not include the number of other bee visits, as a model including that term did not converge (see Table S7 for description of null models). No iterations of the null model reached convergence for *Augochlorella* visits, so this genus was not included in further analyses.

**Table 2:** Coefficient estimates with 95% confidence intervals from the hypothesis (“H”) with the smallest ΔAICc across models for each taxon (in Table 3), where “0” = bee visits are not limited by floral resources; “1” = bee visits are only influenced by the present abundance of local floral resources; “2” = bee visits are influenced by the present abundance of landscape floral resources; “3a” = both the abundance of floral resources in the landscape when foraging begins and any decreases in the abundance of floral resources later in the season influence bee visits; and “3b” = the cumulative abundance of landscape floral resources influences visits

Taxon	H	Scale	Model term	Minimum			Median		
				*β*	2.5%	97.5%	*β*	2.5%	97.5%
All bees	3b	250	CumulativeFR	0.39	–0.08	0.85	0.47	–0.04	0.98
*Andrena*	3a	750	**FirstTimePeriodFR**	**1.79**	**0.30**	**3.28**	**1.66**	**0.19**	**3.13**
			ChangeInFR	–0.37	–1.37	0.63	–0.73	–1.72	0.26
			AllOtherVisits	–0.002	–0.01	0.001	–0.002	–0.01	0.002
*Bombus*	3b	250	CumulativeFR	0.97	–0.02	1.97	0.98	–0.09	2.05
			AllOtherVisits	–0.00003	–0.01	0.01	–0.0001	–0.01	0.01
*Halictus*	3a	750	FirstTimePeriodFR	–0.04	–2.55	2.46	0.23	–2.48	2.93
			**ChangeInFR**	**1.95**	**0.22**	**3.69**	**2.49**	**0.93**	**4.05**
			**TransectFR**	**0.66**	**0.02**	**1.31**	**0.70**	**0.08**	**1.31**
			AllOtherVisits	0.01	–0.004	0.02	0.01	–0.001	0.03
*Lasioglossum*	3a	750	FirstTimePeriodFR	–0.99	–2.47	0.49	–0.98	–2.39	0.44
			**ChangeInFR**	**1.84**	**0.95**	**2.74**	**1.94**	**1.24**	**2.64**
			TransectFR	0.06	–0.22	0.34	0.05	–0.21	0.31
			AllOtherVisits	0.003	–0.004	0.01	0.002	–0.004	0.01
*Megachile*	2b	250	**PresentFR**	**–4.07**	**–7.54**	**–0.60**	**–3.98**	**–7.45**	**–0.51**
			AllOtherVisits	0.01	–0.01	0.02	0.01	–0.01	0.02
*Peponapis*	3a	750	FirstTimePeriodFR	–0.86	–2.14	0.42	–0.69	–2.11	0.74

Note

“Scale” is the size of the radius around a sampling location within which floral resource volume was calculated. Model terms and values in bold represent those for which confidence intervals did not overlap zero, regardless of whether unknown areas in landscapes were assigned the minimum floral resource volume measured across all floral resource‐providing land types (“Minimum”) or the median (“Median”). Estimates of coefficients were calculated by averaging across all median or minimum models for a given hypothesis that had ΔAICc < 7.

**Table 3:** Models of bee visits and corresponding hypotheses, where “Scale” = the landscape scale within which floral resources were measured (250, 500, or 750 meter radius around a sampling location; models were compared at each scale for all taxa), “LL” = log likelihood, and hypotheses (“H”): “0” = bee visits are not limited by floral resources; “1” = bee visits are only influenced by the present abundance of local floral resources; “2” = bee visits are influenced by the present abundance of landscape floral resources (hypotheses 2a and 2b were not assessed during the model selection stage, as this required determining whether the relationship between bee visits and floral resources was positive or negative); “3a” = both the abundance of floral resources in the landscape when foraging begins and any decreases in the abundance of floral resources later in the season influence bee visits; and “3b” = the cumulative abundance of landscape floral resources from when foraging begins influences bee visits

Taxon	H	Scale	Minimum			Median			Total
			LL	AICc	ΔAICc	LL	AICc	ΔAICc	ΔAICc
All bees	3b	250	–730	1,476	0.00	–732	1,476	0.00	0.00
	1	–	–732	1,476	0.15	–732	1,476	0.02	0.17
	2	250	–731	1,476	0.13	–731	1,476	0.52	0.65
	0	–	–733	1,477	1.26	–733	1,477	1.12	2.39
*Andrena*	3a	750	–212	439	0.00	–212	438	0.00	0.00
	3a	500	–213	440	0.72	–213	440	1.70	2.42
	2	250	–214	441	1.80	–214	441	2.62	4.42
	3a	250	–213	441	1.75	–213	441	2.96	4.70
	0	–	–215	441	1.99	–215	441	2.84	4.83
*Bombus*	3b	250	–441	894	0.00	–441	894	0.00	0.00
	3b	750	–441	894	0.54	–442	896	1.47	2.01
	0	–	–442	895	1.77	–442	895	1.09	2.87
	2	250	–441	895	1.47	–442	896	2.15	3.62
*Halictus*	3a	750	–230	479	1.12	–228	475	0.00	1.12
	1	–	–233	478	0.59	–233	478	3.06	3.64
	3a	250	–229	478	0.00	–232	480	4.48	4.48
	2	750	–234	480	2.71	–231	478	2.70	5.42
	2	250	–232	479	1.31	–231	479	4.25	5.56
	0	–	–235	480	2.89	–235	480	5.36	8.26
	3b	750	–232	481	3.93	–233	480	5.21	9.15
	3b	500	–233	480	2.53	–235	483	7.53	10.06
	3b	250	–233	480	2.52	–235	484	8.82	11.34
*Lasioglossum*	3a	750	–315	644	0.00	–312	639	0.00	0.00
	3a	500	–316	647	3.12	–316	648	8.65	11.78
	3b	500	–318	649	4.48	–319	650	11.06	15.55
	3b	750	–318	649	4.59	–319	650	11.07	15.66
	3a	250	–318	651	6.47	–318	650	11.38	17.84
	0	–	–320	651	6.60	–320	651	12.06	18.66
*Megachile*	2	250	–68.7	153	0.00	–68.5	153	1.00	1.00
	2	750	–69.5	155	1.64	–68.0	152	0.00	1.64
	0	–	–71.8	155	1.41	–71.8	155	2.79	4.20
	3a	750	–69.3	157	3.68	–67.2	153	0.97	4.64
	2	500	–71.1	156	2.34	–69.3	154	2.55	4.89
*Peponapis*	3a	750	–181	378	0.00	–188	388	1.60	1.60
	0	–	–188	387	8.30	–188	387	0.00	8.30

Note

Models shown are those with smaller ΔAICc than the null model (H = “0”) when unknown areas in landscapes were assigned either the minimum (“Minimum”) or the median (“Median”) floral resource volume across all floral resource‐providing land types. Models are listed in order of smallest to largest ΔAICc across models (Total = Minimum + Median).

### 3.4 H1: Bee visits are influenced only by local floral resources

No models for any genera supported the hypothesis that only the present abundance of local floral resources influences local visitation rates. The best models for *Halictus*, which also included landscape floral resources as a predictor, showed that visits were positively related to the abundance of floral resources within transects, but no other taxon exhibited a strong relationship with local floral resources (Table 2).

### 3.5 H2: Bee visits are influenced by the present abundance of landscape floral resources

**Figure 4:**
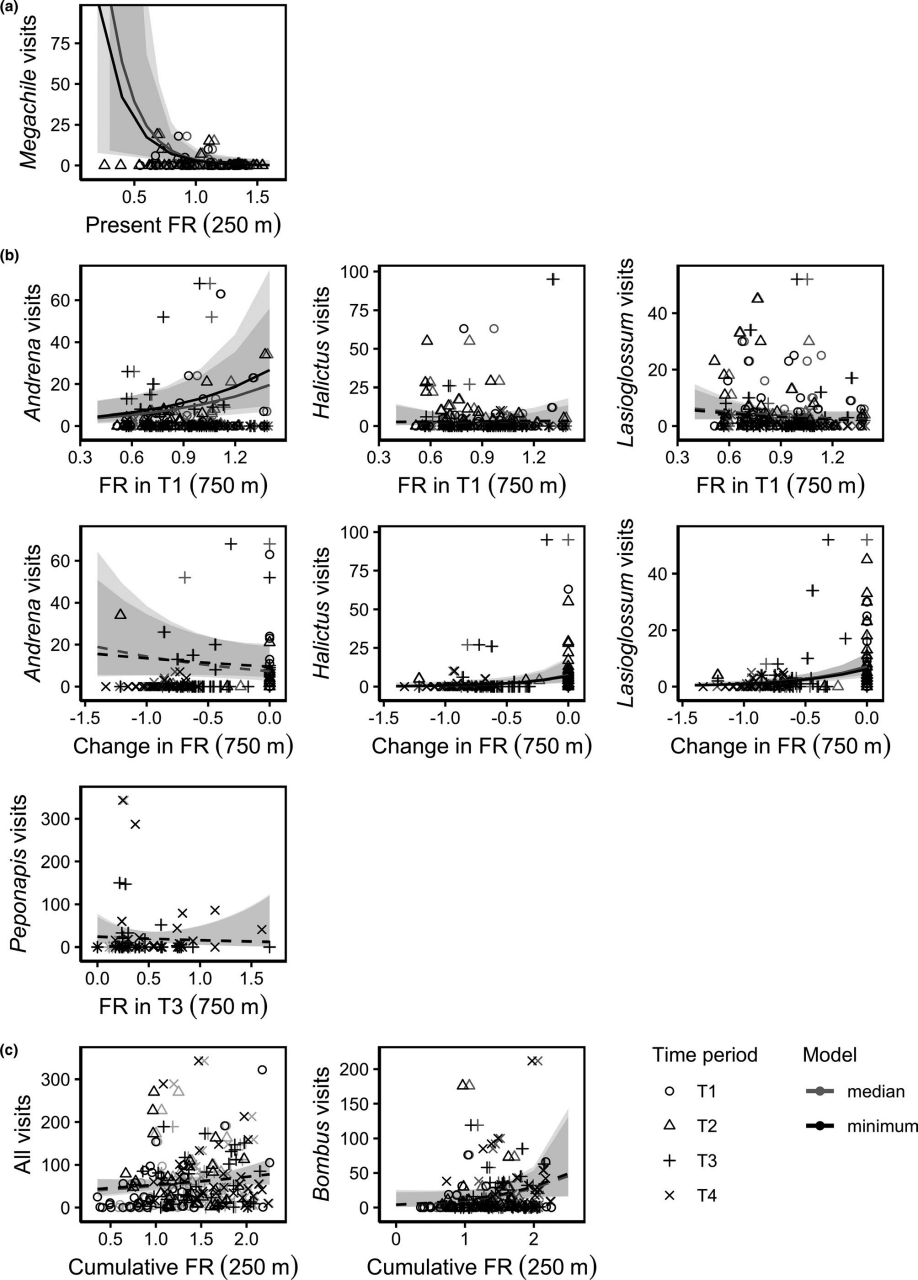
Relationship between the number of bee visits observed and the volume of floral resources (“FR”) measured within the landscape surrounding sampling locations (within a radius of either 250, 500, or 750 m) over four time periods during one season, where T1 = late spring, T2 = early summer, T3 = mid‐summer, and T4 = late summer. Rows represent the hypothesis that was best supported for each taxon: (a) H2 = bee visits are influenced by the present abundance of landscape floral resources; (b) H3a = bee visits are influenced by both the abundance of floral resources in the landscape when foraging begins (T1 for *Andrena*, *Halictus*, and *Lasioglossum*, and T3 for *Peponapis*) and any decreases in the abundance of floral resources later in the season (n.b. this relationship was not examined for *Peponapis* because none of the sampling locations for this taxon experienced declining floral resources from T3 to T4); (c) H3b = bee visits are influenced by the cumulative abundance of landscape floral resources from when foraging begins. Lines represent the predicted marginal effect of floral resource volume on bee visits for a given taxon, where predicted values are conditioned on the zero‐inflation component of models and incorporate uncertainty from random effects. Solid lines represent significant relationships, and dashed lines represent nonsignificant relationships. Shaded areas represent 95% confidence intervals around predicted marginal effects. Relationships are presented from models presented in Table 2, where floral resources were calculated with all unknown areas in a given radius assigned the median volume of floral resources measured across all known resource‐providing land types during a given time period (“median” model) and the minimum volume (“minimum” model)

Visits from *Megachile* best supported the hypothesis that the present abundance of floral resources within the landscape is the main influence on bee visits (Table 3). Specifically, *Megachile* models supported hypothesis 2b that bee visits are not limited by floral resources, but instead are dispersed across landscapes with higher floral resource abundance; visits were negatively associated with the present volume of floral resources measured within a 250 m radius (Table 2, Figure 4a).

### 3.6 H3: Bee visits are influenced by the previous abundance of landscape floral resources

The number of visits observed from all bees, and from *Bombus*, *Andrena*, *Halictus*, *Lasioglossum*, and *Peponapis* individually, best supported the hypothesis that bee visits were influenced by the previous abundance of landscape floral resources (Table 3).

Following hypothesis 3a that both the abundance of floral resources in the landscape when foraging begins and any decreases in the abundance of floral resources later in the season influence bee visits, *Andrena*, *Halictus*, and *Lasioglossum* visits were best described by models that included the volume of floral resources measured within a 750 m radius when foraging first began (in late spring) and the negative change in floral resource volume measured in subsequent time periods. *Andrena* visits were more numerous in landscapes with more abundant late spring floral resources but were not strongly related to the change in floral resources (Table 2, Figure 4b). Although no strong relationship was observed between *Halictus* or *Lasioglossum* visits and late spring floral resources, fewer visits from both genera were observed on average in landscapes that experienced greater decreases in floral resources (Table 2, Figure 4b). *Peponapis* visits were also best predicted by the volume of floral resources within a 750 m radius at the start of the foraging season (mid‐summer) when unknown areas were assigned the minimum floral resource volume (Table 3); however, this negative relationship was weak (Table 2, Figure 4b). Sampling locations where *Peponapis* visits were observed did not experience any declines in floral resource volume between mid‐ and late summer, so the effect of floral resource reductions was not examined.

All bee visits, and *Bombus* visits individually, both supported hypothesis 3b, that the cumulative abundance of landscape floral resources most strongly influences bee visits. Visits were best predicted by models that included the cumulative floral resource volume measured within a 250 m radius (Table 3). All bee visits and *Bombus* visits were positively associated with cumulative floral resources, but neither relationship was significant (Table 2, Figure 4c).

## 4 DISCUSSION

We found that, for the bee community as a whole, the cumulative abundance of floral resources in previous months within the season best predicted visitation rates. However, this relationship was not strong, and it varied among genera. Individual bee taxa exhibit unique combinations of foraging distances, foraging periods, and numbers of broods produced per season, all of which can influence their responses to changes in landscape floral resource abundance over a season. Our research acknowledged these taxonomic differences by assuming each bee genus observed would respond at different spatial and temporal scales to the abundance of floral resources in a landscape. By doing this, we were able to document important differences in the ways in which each genus was influenced by the spatial and temporal availability of floral resources.

Floral resources in the landscape over preceding months were an important predictor of bee activity for several genera. High floral resource abundance at the start of foraging was important for solitary bees that produce only one brood per season (*Andrena* and *Peponapis*), while high floral resource abundance in all previous months was positively related to the number of visits for the most social bees that produced multiple broods per season (*Bombus*). Stable floral resource abundance over previous months was positively associated with the number of visits for bees that were less social and produced fewer broods per season (*Lasioglossum* and *Halictus*). However, for *Megachile*, the provision of stable floral resources in preceding months did not appear to influence bee visits. For this solitary genus, the present abundance of floral resources was negatively related to local bee visits, possibly because of a dilution of pollinators across high floral resource landscapes. The number of visits by other bees was not a strong predictor of visitation rates for any genera, suggesting that competition among bee genera is not a primary driver of visitation patterns, at least at the scale of our study.

Much of the previous research on wild bees responding to floral resources in agricultural landscapes has found that a higher abundance of floral resources is associated with larger populations, higher densities, or greater numbers of visits (Kovács‐Hostyánszki et al., 2013; Mallinger et al., 2016; Mandelik et al., 2012; Martins et al., 2018). In our study, the number of visits by *Bombus* was best predicted by cumulative landscape floral resources, in line with the hypothesis that bees producing multiple broods in a season can increase population sizes within one season with access to more floral resources through time. This finding agrees with previous research examining *B*. *vosnesenskii* colony responses to floral resources in agricultural landscapes, which found that the production of males and workers was more positively related to high early‐season floral resource abundance in the surrounding landscape than to late‐season floral resources (Williams et al., 2012). Bumble bees represent some of the most common and important pollinators for both wildflowers and crops in the Northern Hemisphere, but many species are declining (Goulson et al., 2008). Though the positive relationship between *Bombus* visits and cumulative floral resources was not strong, our results suggest that early‐season floral resources in agricultural landscapes could promote high bumble bee visitation rates later in the season, and potentially maintain or increase colony sizes over a season.

For the other two (partially) social genera, *Halictus* and *Lasioglossum*, our findings suggest that these bees were likely producing a single brood per season rather than multiple broods and therefore were able to maintain (but not increase) their population sizes when floral resources were consistent or increased over time. In the region this study was conducted, both *Halictus* and *Lasioglossum* include eusocial species that produce multiple broods per season and solitary species that only produce a single brood (Mitchell, 1960; Packer et al., 2007). Given that these genera responded as expected for single‐brood‐producing bees, we infer either that predominantly solitary species were present in our study locations, or that the more social species in this region were producing too few brood per season to exhibit a strong response to the cumulative abundance of floral resources. Both the degree of sociality and the number of broods produced vary geographically within single species of *Halictus* and *Lasioglossum*, with a general pattern of more solitary bees and fewer broods being produced at higher elevations and latitudes (Davison & Field, 2016; Richards & Packer, 1995; Wcislo, 1997). In our study locations, most *Halictus* and *Lasioglossum* species are closer to their northern range limit (Mitchell, 1960), making it likely that solitary life histories predominate.

The strictly solitary bees examined in this study (*Andrena*, *Megachile*, and *Peponapis*) exhibited differing responses to the temporal pattern of floral resource abundance. We observed *Megachile* foraging during the second, third, and fourth time periods, but a number of the species in this region have more restricted foraging periods (Mitchell, 1962; Sheffield et al., 2011). These shorter foraging periods in *Megachile* species likely explain why this genus responded only to the present abundance of floral resources. The single local species of *Peponapis*, *P. pruinosa*, is a specialist on pollen in the Cucurbitaceae family and has a short foraging period synchronized with its flowering in southern Ontario (Willis & Kevan, 1995). Given the prevalence of species with short flight periods in our study area, the foraging periods of many individual species were likely too short to respond to fluctuations in floral resources at the roughly monthly scale we considered. Future research should focus on examining how fluctuations in floral resources over shorter temporal scales (e.g., weekly) influence bee activity, which would allow for development of agricultural landscapes that specifically benefit those species with short flight periods.

*Andrena* were observed foraging over the entire season; this genus comprises 75 species in eastern Canada (Packer et al., 2007), many of which are active as adults for just one or two months, and are early‐season foragers (LaBerge, 1986; Larkin et al., 2008). We found that floral resource abundance at the start of the season was most important for this genus, which could indicate that our observations were dominated by *Andrena* species that either had long foraging periods or were early‐season foragers. Alternatively, habitats like forest or apple orchards that only provided floral resources early in the season may also provide high‐quality nesting habitat (e.g., untilled soil) for ground‐nesting bees like *Andrena*. In general, availability of nesting habitat for wild bees could be positively or negatively correlated with floral resource abundance (Sardiñas et al., 2016), and our study design does not allow us to disentangle any potentially confounded effects of nesting habitat and floral resources. We suggest follow‐up research should examine seasonal changes in both floral resource and nesting habitat availability to determine how specific habitat types can benefit or hinder wild bees.

*Megachile* and *Peponapis* were the only taxa that showed no positive relationship between visitation rates and floral resource abundance at any temporal or spatial scale. *Peponapis* visits were best predicted by models that included the previous abundance of floral resources, but this genus experienced either an increase or no change in the abundance of floral resources from mid‐ to late summer and had a weakly negative relationship with the abundance of floral resources in mid‐summer. *Megachile* visits were generally fewer in landscapes that had plentiful floral resources. Several studies have previously found that abundant floral resources can decrease bee density on crops, either through dilution of pollinators across a landscape (Holzschuh et al., 2016; Kovács‐Hostyánszki et al., 2013), or through distraction of pollinators from crops to other resource‐rich areas (Lander et al., 2011; Nicholson et al., 2019). This may indicate that the landscapes used in our study generally provided a large amount of floral resources outside the local sampling areas during the *Megachile* flight season (early to late summer), perhaps because we selected sampling locations based on their proximity to farms growing fruit or vegetables. Population sizes for solitary bees that produce a single brood per season should also be strongly limited by the amount of floral resources available in the previous year, which would be used to produce the generation foraging in the current year. We have no data on the previous year's floral resources, but this may have been the primary factor limiting bee populations, especially for species that only forage for a few weeks in a season.

Differences in body size can contribute to differences in the maximum foraging ranges of bee taxa (Greenleaf et al., 2007) and thus to availability of floral resources to bees within the landscape surrounding sampling locations. Individual species can vary greatly in body size (Mitchell, 1960), but the average body size across a genus did not always correlate with the spatial scales at which landscape floral resources were most relevant. Visits for smaller‐bodied genera like *Andrena* (body length 4–15 mm; Mitchell, 1960), *Halictus* (7–13 mm; Mitchell, 1960), and *Lasioglossum* (3.5–10 mm; Mitchell, 1960) were best predicted by floral resources measured within a 750 m radius, while the relatively large‐bodied genera *Bombus* (body length 5–28 mm; Laverty & Harder, 1988) and *Megachile* (6–25 mm; Sheffield et al., 2011) were best predicted by floral resources within a 250 m radius. The spatial arrangement of both floral resources and nesting habitat in a landscape are likely the more relevant predictors of how far most bees actually forage (Zurbuchen et al., 2010). The spatial scales we have determined as the best predictors of bee visits may represent the upper end of foraging areas used by bees in the majority of landscapes that we examined, with many bees actually foraging in smaller areas of landscapes with more densely packed resources. This might be why we observed positive relationships between floral resources and *Halictus* and *Lasioglossum* visits at both local and landscape scales. Although both of these genera appeared to be limited by the abundance of floral resources within a 750 m radius, transects with high floral resources were likely located in high density resource patches relative to the surrounding landscape, resulting in more local bee activity.

By teasing apart the relationships between individual bee genera and floral resources in the landscape, we discovered a diversity of responses among taxonomic groups, highlighting potential problems with lumping all non‐*Bombus* bees into a single functional group, or with examining responses of the entire bee community to floral resources. Our research highlights the importance of not only the current floral resource landscape but also the floral resources present earlier in the season. This information can help determine how to configure agricultural landscapes in a way that promotes bee population persistence and growth and, thus, increases the pollination services crops receive. Our findings suggest that bees with longer flight periods likely benefit from continuous, consistent provision of floral resources throughout a single season, and high floral resource abundance early in the season. However, the number of bee visits observed may not be a good proxy for bee population sizes. An important next step will be to determine how across‐year patterns in visitation rates change with spatiotemporal fluctuations in floral resources, particularly for single‐brood species which can only respond positively to floral resource availability over a longer timescale. Though we observed fewer visits from some solitary bees with short foraging periods in landscapes with abundant floral resources, this pattern should not hold across years: If other factors are not limiting, more abundant floral resources in landscapes should yield higher bee abundances in subsequent years.

## CONFLICT OF INTEREST

None declared.

## AUTHOR CONTRIBUTION

**Jessica Marie Guezen:** Conceptualization (equal); Data curation (lead); Formal analysis (lead); Funding acquisition (equal); Methodology (equal); Resources (equal); Validation (lead); Writing‐original draft (lead); Writing‐review & editing (lead). **Jessica R. K. Forrest:** Conceptualization (equal); Formal analysis (supporting); Funding acquisition (equal); Methodology (equal); Resources (equal); Supervision (lead); Validation (supporting); Writing‐original draft (supporting); Writing‐review & editing (supporting).

## Supporting information

Figure S1

Tables S1–S7

## Data Availability

Floral counts in quadrats, bee visit observations, data used for modeling, and shapefiles for all land types and sampling locations are available on Dryad (https://doi.org/10.5061/dryad.zs7h44j7m).
