# Supplementary figures and images for: Seasonality of floral resources in relation to bee activity in agroecosystems
Source: Ecol Evol. 2021 Feb 28;11(7):3130–47. doi: 10.1002/ece3.7260 (PMC8019032; doi:10.1002/ece3.7260)

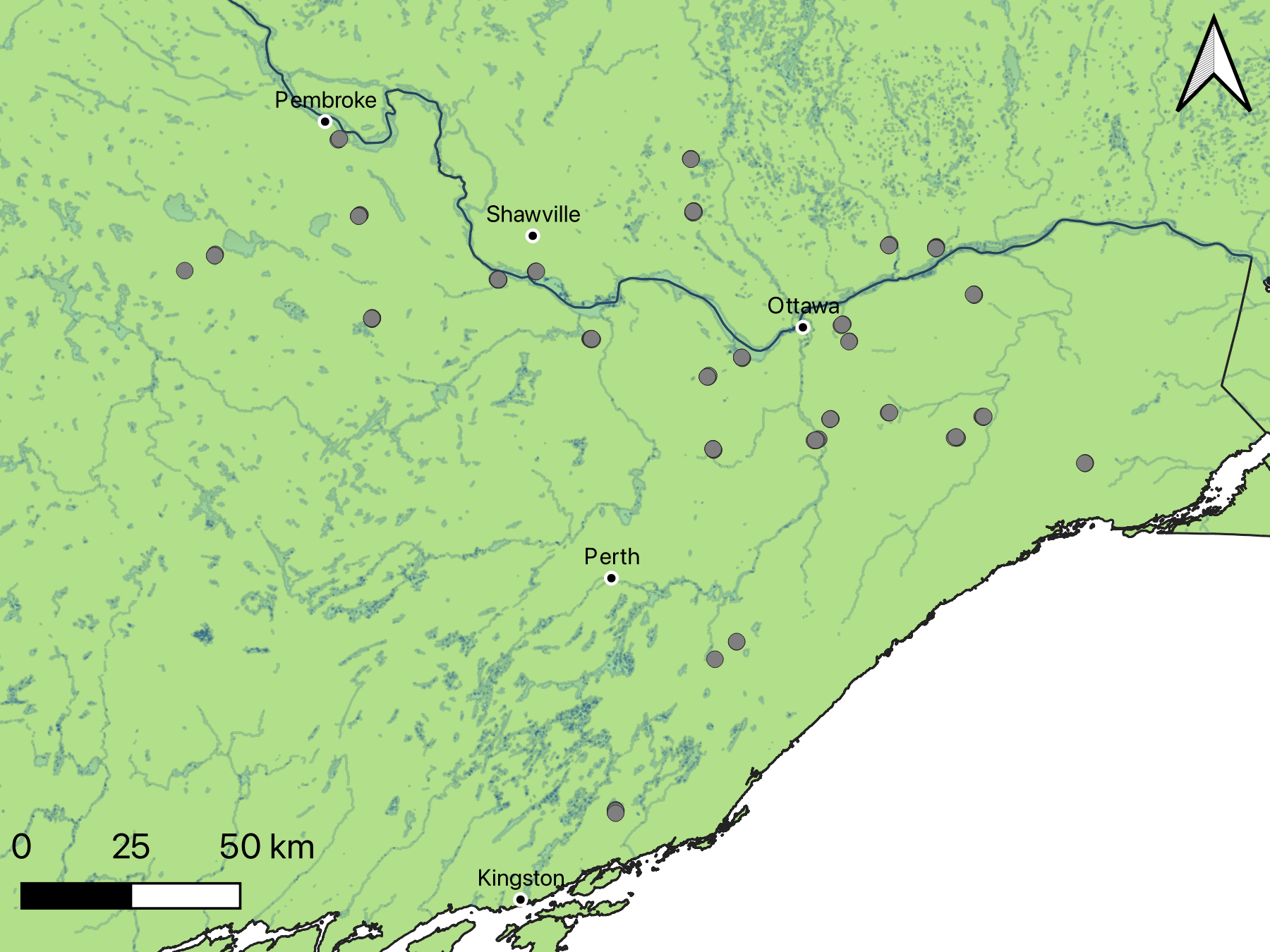

Supplement: Supplementary file 1 — Figure S1 [file ECE3-11-3130-s002.png]
