# Supplementary material for: Seasonality of floral resources in relation to bee activity in agroecosystems
Source: Ecol Evol. 2021 Feb 28;11(7):3130–47. doi: 10.1002/ece3.7260 (PMC8019032; doi:10.1002/ece3.7260)
Supplement: Supplementary file 2 — Tables S1–S7 [file ECE3-11-3130-s001.docx]

**Table S1** Land-types used to quantify landscape structure and floral resources around sampling locations. The range in proportion of each land type across all sampling periods is represented as the percent of total area within a 750-m radius around each sampling location. Areas with water, exposed or barren land, and urban or developed land were not digitized, and the proportions are taken from raster images from Agriculture and Agri-Food Canada’s 2016 Annual Crop Inventory. Soybean, unknown crop, hedgerow, and potentially resource-providing crop land types were not assessed for floral resources, and a range is presented for values assigned in models run, from the minimum to the median floral resource volume from all resource-providing land types in a given spatial scale and sampling period.

| Land type | Description | Area in 750-m radius (%) | Median floral resources by sampling period (cm^3^/m^2^) |
| --- | --- | --- | --- |
| **Non-resource land** | | | |
| Exposed land and barren | Category from Agriculture and Agri-Food Canada’s 2016 Annual Crop Inventory (not manually digitized) | 0.05–10 | 0.00 |
| Urban and developed | Category from Agriculture and Agri-Food Canada’s 2016 Annual Crop Inventory (not manually digitized) | 0.00–44 | 0.00 |
| Water | Category from Agriculture and Agri-Food Canada’s 2016 Annual Crop Inventory (not manually digitized) | 0.00–20 | 0.00 |
| Cereal crop | Barley, millet, oats, rye, spelt, triticale, wheat | 0.002 –15 | 0.00 |
| Corn | Only known to be a pollen resources for *Apis mellifera* (Danner et al. 2014, Requier et al. 2015) | 0.3–38 | 0.00 |
| Herbs, field vegetables | Non-flowering or harvested before flowering | 0.02–5.4 | 0.00 |
| **Resource-providing land** | | | |
| Semi-natural | Grass land, shrub land | 0.4–35 | 0.20 (late spring)  2.82 (early summer)  1.00 (mid-summer)  1.53 (late summer) |
| Forage | *Medicago sativa*, hay, pasture, *Trifolium* spp. | 1.7–60 | 3.67 (late spring)  3.49 (early summer)  4.23 (mid-summer)  1.49 (late summer) |
| Forest |  | 4.6–87 | 0.14 (late spring)  0.55 (early summer) |
| Apple | *Malus* *pumila* | 0.05–4.2 | 41.6 (late spring) |
| Asparagus | *Asparagus officinalis* | 0.01–0.5 | 0.84 (early summer) |
| Bean | *Phaseolus vulgaris* | 0.006–0.5 | 0.08 (mid-summer) |
| Cucumber | *Cucumis sativus* | 0.003–0.02 | 0.28 (late summer) |
| Melon | *Cucumis melo* | 0.01–0.8 | 0.24 (mid-summer) |
| Potato | *Solanum tuberosum* | 0.26–0.29 | 0.75 (early summer)  0.88 (mid-summer) |
| Raspberry | *Rubus idaeus*, *R. strigosus*,  *R. occidentalis* | 0.002–0.9 | 2.31 (early summer) |
| Squash | *Cucurbita* spp. | 0.01–7.1 | 0.37 (early summer)  0.66 (mid-summer)  1.09 (late summer) |
| Strawberry | *Fragaria* × *ananassa* | 0.5–3.5 | 12.6 (late spring)  13.0 (early summer) |
| Sunflower | *Helianthus annuus* | 0.005–0.05 | 31.2 (early summer)  33.8 (mid-summer)  36.3 (late summer) |
| Watermelon | *Citrullus lanatus* | 0.01–0.2 | 0.14 (mid-summer) |
| **Unknown land** | | | |
| Soybean | Potentially resource-providing crop | 1.5–55 | 0.00 (late spring)  0–1.57 (early summer)  0–0.84 (mid-summer)  0.00 (late summer) |
| Unknown crop |  | 0.08–21 | 0–1.30 (late spring)  0–1.57 (early summer)  0–0.84 (mid-summer)  0–0.54 (late summer) |
| Hedgerow | Areas bordering agricultural fields with shrubs or trees | 0.05–6.8 | 0–1.30 (late spring)  0–1.57 (early summer)  0–0.84 (mid-summer)  0–0.54 (late summer) |
| Other potentially resource-providing crops | *Brassica rapa*, *B. napus*, *B. juncea*, *Capsicum annuum*, *Fagopyrum esculentum*, *Lycopersicon esculentum*, *Physalis* spp., *Pisum sativum*, *Prunus avium*, *Pyrus communis*, *Rubus fruticosus* | 0.002–1.1 | 0–1.30 (late spring)  0–1.57 (early summer)  0–0.84 (mid-summer)  0–0.54 (late summer) |

**Table S2** List of bee species identified across all sampling locations.

| Genus | Species |
| --- | --- |
| *Andrena* | *Andrena canadensis* Dalla Torre, 1896  *Andrena carlini* Cockerell, 1901  *Andrena cressonii* Robertson, 1891  *Andrena imitatrix* Cresson, 1872  *Andrena integra* Smith, 1853  *Andrena miserabilis* Cresson, 1872  *Andrena nigrihirta* (Ashmead, 1890)  *Andrena perplexa* Smith, 1853  *Andrena wilkella* (Kirby, 1802) |
| *Apis* | *Apis mellifera* Linnaeus, 1758 |
| *Augochlorella* | *Augochlorella aurata* (Smith, 1853) |
| *Augochlora* | *Augochlora pura* (Say, 1837) |
| *Bombus* | *Bombus bimaculatus* Cresson, 1863  *Bombus borealis* Kirby, 1837  *Bombus impatiens* Cresson, 1863  *Bombus pensylvanicus* (De Geer, 1773)  *Bombus rufocinctus* Cresson, 1863  *Bombus sandersoni* Franklin, 1913  *Bombus ternarius* Say, 1837  *Bombus terricola* Kirby, 1837  *Bombus vagans* Smith, 1854 |
| *Ceratina* | *Ceratina calcarata* Robertson, 1900 |
| *Colletes* | *Colletes simulans armatus* Patton, 1879  *Colletes willistoni* Robertson, 1891 |
| *Halictus* | *Halictus confusus* Smith, 1853  *Halictus ligatus* Say, 1837 |
| *Heriades* | *Heriades carinatus* Cresson, 1864 |
| *Hoplitis* | *Hoplitis pilosifrons* (Cresson, 1864) |
| *Hylaeus* | *Hylaeus modestus* Say, 1837 |
| *Lasioglossum* | *Lasioglossum taylorae* Gibbs, 2010  *Lasioglossum* *versatum* (Robertson, 1902)  *Lasioglossum coriaceum* (Smith, 1853)  *Lasioglossum leucozonium* (Schrank, 1781)  *Lasioglossum zonulum* (Smith, 1848) |
| *Megachile* | *Megachile latimanus* Say, 1823  *Megachile melanophaea* Smith, 1853  *Megachile relativa* Cresson, 1878  *Megachile rotundata* (Fabricius, 1793) |
| *Melissodes* | *Melissodes druriella* (Kirby, 1802)  *Melissodes subillata* LaBerge, 1961 |
| *Osmia* | *Osmia distincta* Cresson, 1864  *Osmia lignaria* Say, 1837 |
| *Peponapis* | *Peponapis pruinosa* (Say, 1837) |
| *Perdita* | *Perdita octomaculata* (Say, 1824) |

**Table S3** Measurements and literature sources of floral dimensions for all non-graminoid flowering species. Floral units describe the scale at which species were counted in quadrats (flower, inflorescence, or capitulum in Asteraceae species). ‘*l*’ is length in mm of corolla opening (or receptacle length for capitula), ‘*w*’ is width in mm of corolla opening (or receptacle width for capitula), ‘*h*’ is height in mm, measured from the receptacle to the longest sexual organ (stamen or pistil), and ‘*V*’ is floral volume in mm^3^, calculated using the formula for volume of an elliptic cylinder ($\text{V}\text{ }\text{=}\text{ }\text{π}\text{ }\text{×}\text{ }\frac{\text{l}}{\text{2}}\text{ }\text{×}\text{ }\frac{\text{w}}{\text{2}}\text{ }\text{×}\text{ }\text{h}$). Inflorescence volume was calculated by multiplying floral volume by the average number of flowers per inflorescence and was left blank when individual flowers were counted rather than inflorescences. Measurements were made on five individuals per species, or values were obtained from literature sources.

| Species | Floral unit | *l* | *w* | *h* | *V* | Inflorescence *V* (mm^3^) | Sources |
| --- | --- | --- | --- | --- | --- | --- | --- |
| *Acer spicatum* | inflorescence | 2.0 | 2.0 | 3.0 | 9.42 | 1649 | (Sullivan 1983) |
| *Achillea millefolium* | inflorescence | 6.0 | 6.0 | 3.4 | 96.1 | 1442 | Measured; <https://nature.ca/aaflora>; <http://www.efloras.org> |
| *Actaea pachypoda* | inflorescence | 2.0 | 2.0 | 5.0 | 15.7 | 298 | (Pellmyr 1985); <http://plants.jstor.org>; <http://www.illinoiswildflowers.info> |
| *Agrimonia gryposepala* | flower | 5.0 | 5.0 | 5.0 | 98.2 |  | <http://plants.jstor.org> |
| *Anthemis arvensis* | capitulum | 6.2 | 6.2 | 6.4 | 226 |  | Measured |
| *Asclepias syriaca* | inflorescence | 2.0 | 2.0 | 2.0 | 6.28 | 126 | Measured |
| *Asparagus officinalis* | flower | 1.2 | 1.2 | 4.8 | 6.13 |  | Measured |
| *Barbarea vulgaris* | inflorescence | 1.0 | 1.0 | 2.0 | 1.57 | 12.6 | Measured; <http://www.discoverlife.org> |
| *Capsella bursa-pastoris* | inflorescence | 2.0 | 2.0 | 2.0 | 6.28 | 37.7 | (Nave et al. 2016); <http://www.efloras.org>; <http://www.discoverlife.org> |
| *Cardamine bulbosa* | flower | 1.8 | 1.8 | 1.0 | 2.98 |  | Measured |
| *Cardamine diphylla* | flower | 2.0 | 2.8 | 7.0 | 29.5 |  | Measured |
| *Centaurea* sp*.* | capitulum | 8.0 | 8.0 | 22.0 | 1143 |  | Measured |
| *Cerastium fontanum* | flower | 1.0 | 1.0 | 3.6 | 2.83 |  | Measured |
| *Chelidonium* sp. | flower | 5.0 | 5.0 | 8.0 | 157 |  | (Kang et al. 1991); <http://plants.jstor.org> |
| *Cichorium intybus* | capitulum | 4.6 | 4.6 | 9.4 | 161 |  | Measured |
| *Citrullus lanatus* | flower | 5.0 | 5.0 | 5.2 | 102 |  | Measured |
| *Coptis trifolia* | flower | 7.6 | 7.6 | 4.2 | 197 |  | Measured |
| *Cornus canadensis* | flower | 7.8 | 7.8 | 4.0 | 193 |  | Measured |
| *Cornus sericea* | inflorescence | 1.0 | 1.0 | 5.4 | 4.24 | 106 | Measured; <http://www.discoverlife.org> |
| *Cucumis melo* | flower | 7.5 | 7.5 | 4.5 | 199 |  | <http://www.efloras.org> |
| *Cucumis sativus* | flower | 2.0 | 2.0 | 6.2 | 19.5 |  | Measured |
| *Cucurbita pepo* (female zucchini) | flower | 10.2 | 10.2 | 16.6 | 1353 |  | Measured |
| *Cucurbita pepo* (male zucchini) | flower | 8.4 | 8.4 | 16.0 | 934 |  | Measured |
| *Cucurbita pepo* (zucchini) | flower | 9.3 | 9.3 | 16.3 | 1107 |  | Measured |
| *Cucurbita* sp. (female squash) | flower | 13.8 | 13.8 | 19.8 | 3091 |  | Measured |
| *Cucurbita* sp. (male squash) | flower | 10.4 | 8.4 | 15.8 | 1127 |  | Measured |
| *Cucurbita* sp. (squash*)* | flower | 12.1 | 11.1 | 17.8 | 1878 |  | Measured |
| *Cucurbita* spp*.* | flower | 10.7 | 10.2 | 17.1 | 1461 |  | Measured |
| *Cucurbita* spp. (female) | flower | 12.0 | 12.0 | 18.2 | 2058 |  | Measured |
| *Cucurbita* spp. (male*)* | flower | 9.4 | 8.4 | 15.9 | 986 |  | Measured |
| *Daucus carota* | inflorescence | 2.0 | 2.0 | 1.0 | 3.14 | 358 | Measured; (Sivinski et al. 2011) |
| *Echium vulgare* | flower | 2.4 | 3.8 | 9.2 | 65.5 |  | Measured |
| *Erigeron canadensis* | inflorescence | 1.0 | 1.0 | 3.0 | 2.36 | 1275 | Measured |
| *Erigeron philadelphicus* | capitulum | 7.6 | 7.6 | 3.4 | 157 |  | Measured |
| *Erigeron* sp*.* | capitulum | 3.55 | 3.55 | 12.6 | 52.5 |  | Measured |
| *Erysimum cheiranthoides* | inflorescence | 1.0 | 1.0 | 3.0 | 2.36 | 18.8 | (Idris and Grafius 1995); <http://www.discoverlife.org> |
| *Erythronium americanum* | flower | 3.0 | 3.0 | 11.0 | 77.8 |  | Measured |
| *Euthamia graminifolia* | inflorescence | 1.0 | 1.0 | 1.0 | 0.79 | 20.4 | (Harder 1985); <http://plants.jstor.org> |
| *Fagopyrum esculentum* | inflorescence | 2.0 | 2.0 | 2.0 | 6.28 | 980 | (Cawoy et al. 2006, 2009) |
| *Fragaria* × *ananassa* | flower | 7.8 | 7.8 | 6.2 | 304 |  | Measured |
| *Fragaria virginiana* | flower | 3.0 | 3.0 | 2.4 | 26.4 |  | Measured |
| *Galinsoga quadriradiata* | capitulum | 2.0 | 2.0 | 2.0 | 0.01 |  | (Warwick and Sweet 1983) |
| *Galium mollugo* | inflorescence | 1.0 | 1.0 | 1.0 | 0.79 | 14.1 | <https://weedecology.css.cornell.edu>; <http://www.discoverlife.org> |
| *Galium palustre* | inflorescence | 1.0 | 1.0 | 1.0 | 0.79 | 5.50 | <https://weedecology.css.cornell.edu>; <http://www.luontoportti.com> |
| *Geum aleppicum* | flower | 6.0 | 6.0 | 8.0 | 226 |  | <http://symbiota.math.wisc.edu> |
| *Glechoma hederacea* | flower | 1.0 | 1.0 | 9.8 | 7.70 |  | Measured |
| *Helianthus annuus* | capitulum | 35.4 | 35.4 | 20.7 | 27435 |  | Measured |
| *Hieracium caespitosum* | capitulum | 7.4 | 7.4 | 3.4 | 153 |  | Measured |
| *Hieracium pilosella* | capitulum | 10.4 | 10.4 | 6.4 | 538 |  | Measured |
| *Lactuca* sp*.* | capitulum | 2.0 | 2.0 | 3.0 | 9.42 |  | <http://www.naturemanitoba.ca> |
| *Leucanthemum vulgare* | capitulum | 10.5 | 10.5 | 3.5 | 311 |  | Measured |
| *Linaria vulgaris* | flower | 2.4 | 1.0 | 20.6 | 39.1 |  | Measured |
| *Lotus corniculatus* | flower | 4.73 | 4.73 | 12.0 | 211 |  | <http://ucjeps.berkeley.edu/eflora> |
| *Maianthemum canadense* | inflorescence | 1.0 | 1.0 | 1.0 | 0.79 | 14.9 | <http://plants.jstor.org> |
| *Malus pumila* | flower | 9.2 | 9.4 | 9.8 | 674 |  | Measured |
| *Malva neglecta* | flower | 1.9 | 1.9 | 4.25 | 12.0 |  | (Nave et al. 2016) |
| *Medicago lupulina* | inflorescence | 1.0 | 1.0 | 1.0 | 0.79 | 11.8 | Measured; <http://www.efloras.org> |
| *Medicago sativa* | inflorescence | 1.0 | 1.0 | 5.0 | 3.93 | 68.7 | Measured; (Winkler et al. 2009); <http://www.efloras.org> |
| *Mitella diphylla* | flower | 1.0 | 1.0 | 3.0 | 2.36 |  | Measured |
| *Oenothera biennis* | flower | 1.0 | 1.0 | 6.4 | 5.03 |  | Measured |
| *Oxalis stricta* | flower | 1.0 | 1.0 | 3.5 | 2.75 |  | Measured |
| *Phaseolus vulgaris* | flower | 2.8 | 2.8 | 7.6 | 48.2 |  | Measured |
| *Physalis heterophylla* | flower | 2.8 | 2.8 | 9.0 | 58.1 |  | Measured |
| *Plantago lanceolata* | inflorescence | 1.0 | 1.0 | 2.0 | 6.28 | 452 | (Cavers et al. 1980, Van Damme 1984) |
| *Potentilla argentea* | flower | 2.4 | 2.4 | 1.2 | 6.13 |  | Measured |
| *Prunella vulgaris* | flower | 1.2 | 1.6 | 7.0 | 11.2 |  | Measured |
| *Ranunculus acris* | flower | 5.8 | 5.8 | 2.8 | 80.9 |  | Measured |
| *Ranunculus recurvatus* | flower | 1.6 | 1.6 | 2.6 | 6.28 |  | Measured |
| *Rubus allegheniensis* | flower | 11.6 | 11.6 | 4.6 | 489 |  | Measured |
| *Rubus* sp. (raspberry*)* | flower | 6.4 | 6.4 | 6.0 | 202 |  | Measured |
| *Rudbeckia hirta* | capitulum | 15.0 | 15.0 | 3.6 | 636 |  | <http://www.efloras.org> |
| *Silene vulgaris* | flower | 3.2 | 3.2 | 15.6 | 133 |  | Measured |
| *Sisyrinchium montanum* | flower | 1.0 | 1.0 | 4.5 | 3.53 |  | Measured |
| *Solanum tuberosum* | flower | 2.8 | 2.8 | 9.0 | 57.3 |  | Measured |
| *Solidago canadensis* | inflorescence | 1.0 | 1.0 | 1.0 | 0.79 | 26.7 | Measured; (Harder 1985) |
| *Solidago* sp*.* | inflorescence | 1.0 | 1.0 | 1.0 | 0.79 | 26.7 | Measured; (Harder 1985) |
| *Sonchus arvensis* | capitulum | 10.5 | 10.5 | 22 | 1905 |  | (Lemna and Messersmith 1990) |
| *Symphotrichum lanceolatum* | capitulum | 4.0 | 4.0 | 6.0 | 75.4 |  | (Chmielewski and Semple 2001) |
| *Taraxacum* agg*.* | capitulum | 19.6 | 19.6 | 8.4 | 3050 |  | Measured |
| *Trifolium aureum* | inflorescence | 1.0 | 1.0 | 7.0 | 5.50 | 179 | <http://www.efloras.org> |
| *Trifolium pratense* | inflorescence | 1.0 | 1.0 | 11.0 | 8.64 | 432 | (Winkler et al. 2009); <http://www.efloras.org> |
| *Trifolium repens* | inflorescence | 1.0 | 1.0 | 2.0 | 1.57 | 55.0 | (Nave et al. 2016); <http://www.efloras.org> |
| *Trillium erectum* | flower | 8.0 | 8.0 | 9.0 | 452 |  | <http://www.efloras.org> |
| *Trillium grandiflorum* | flower | 5.4 | 5.6 | 11.0 | 292 |  | Measured |
| *Verbena hastata* | inflorescence | 1.0 | 1.0 | 2.0 | 1.57 | 8.80 | Measured |
| *Veronica serpyllifolia* | flower | 1.0 | 1.0 | 1.13 | 0.88 |  | Measured |
| *Veronica* sp*.* | flower | 1.04 | 1.04 | 1.04 | 0.88 |  | Measured |
| *Vicia cracca* | inflorescence | 3.0 | 3.0 | 6.2 | 43.8 | 1972 | Measured; <http://linnet.geog.ubc.ca/Atlas> |
| *Viola pubescens* | flower | 1.0 | 1.0 | 4.5 | 3.53 |  | Measured |

**Table S4** Literature values for nectar production in 46 flowering species. “Nectar unit” is the scale at which nectar was collected from species, with capitulum measurements for members of Asteraceae and individual flower measurements for all other species. “Source species” were used when nectar production values were only available for morphologically similar species within the same genus and is left blank when the species itself was used.

| Species | Nectar (μg/day) | Nectar unit | Source species | Source |
| --- | --- | --- | --- | --- |
| *Acer spicatum* | 77.0 | flower |  | (Heinrich 1976) |
| *Achillea millefolium* | 38.1 | capitulum |  | (Holl 1995, Hicks et al. 2016) |
| *Agrimonia gryposepala* | 1.37 | flower | *A. eupatoria* | (Baude et al. 2016) |
| *Anthemis arvensis* | 2880 | capitulum |  | (Schultz and Dlugosch 1999) |
| *Asclepias syriaca* | 1750 | flower |  | (Heinrich 1976) |
| *Barbarea vulgaris* | 158 | flower |  | (Holl 1995) |
| *Capsella bursa-pastoris* | 0.39 | flower |  | (Baude et al. 2016) |
| *Cardamine bulbosa* | 4.50 | flower | *C.* spp. | (Baude et al. 2016) |
| *Centaurea* sp*.* | 1474 | capitulum | *C. nigra* | (Hicks et al. 2016) |
| *Cerastium fontanum* | 26.9 | flower |  | (Baude et al. 2016) |
| *Citrullus lanatus* | 12000 | flower |  | (Taha and Bayoumi 2009) |
| *Cornus sericea* | 68.8 | flower | *C. sanguinea* | (Baude et al. 2016) |
| *Cucurbita* sp. (female squash) | 30810 | flower | *C. pepo* | (Vidal et al. 2006) |
| *Daucus carota* | 7.35 | flower |  | (Baude et al. 2016) |
| *Echium vulgare* | 688 | flower |  | (Hicks et al. 2016) |
| *Erigeron philadelphicus* | 2250 | capitulum |  | (Holl 1995) |
| *Euthamia graminifolia* | 24.0 | capitulum |  | (Heinrich 1976) |
| *Fragaria virginiana* | 42.0 | flower |  | (Heinrich 1976) |
| *Galium mollugo* | 3.58 | flower |  | (Baude et al. 2016) |
| *Geum aleppicum* | 29.8 | flower | *G. urbanum* | (Baude et al. 2016) |
| *Glechoma hederacea* | 94.4 | flower |  | (Baude et al. 2016) |
| *Leucanthemum vulgare* | 515 | capitulum |  | (Hicks et al. 2016) |
| *Linaria vulgaris* | 544 | flower |  | (Baude et al. 2016) |
| *Lotus corniculatus* | 61.8 | flower |  | (Baude et al. 2016) |
| *Malus pumila* | 110 | flower |  | (Baude et al. 2016) |
| *Malva neglecta* | 541 | flower | *M. moschata* | (Hicks et al. 2016) |
| *Medicago lupulina* | 1.63 | flower |  | (Baude et al. 2016) |
| *Medicago sativa* | 146 | flower |  | (Baude et al. 2016) |
| *Oxalis stricta* | 15.5 | flower | *O. acetosella* | (Baude et al. 2016) |
| *Phaseolus vulgaris* | 53.4 | flower | Cultivated bean | (Baude et al. 2016) |
| *Potentilla argentea* | 23.3 | flower | *Potentilla* spp. | (Baude et al. 2016) |
| *Prunella vulgaris* | 139 | flower |  | (Baude et al. 2016) |
| *Ranunculus acris* | 78.8 | flower |  | (Baude et al. 2016) |
| *Rubus allegheniensis* | 894 | flower |  | (Holl 1995) |
| *Rubus* sp. (raspberry) | 1893 | flower | *R. fruticosus* agg. | (Baude et al. 2016) |
| *Silene vulgaris* | 251 | flower |  | (Baude et al. 2016) |
| *Solanum tuberosum* | 27.3 | flower |  | (Baude et al. 2016) |
| *Solidago canadensis* | 28.8 | capitulum |  | (Heinrich 1976) |
| *Sonchus arvensis* | 651 | capitulum |  | (Baude et al. 2016) |
| *Symphotrichum lanceolatum* | 1116 | capitulum |  | (Chmielewski and Semple 2001) |
| *Taraxacum* agg. | 2137 | capitulum |  | (Hicks et al. 2016) |
| *Trifolium pratense* | 117 | flower |  | (Baude et al. 2016) |
| *Trifolium repens* | 49.0 | flower |  | (Baude et al. 2016) |
| *Veronica serpyllifolia* | 2.83 | flower |  | (Baude et al. 2016) |
| *Vicia cracca* | 484 | flower |  | (Baude et al. 2016) |
| *Viola pubescens* | 125 | flower | *Viola* spp. | (Holl 1995, Baude et al. 2016) |

**Table S5** Literature values for pollen volume in 33 flowering species. Pollen volume was provided in literature sources directly or was calculated from literature values of pollen grain counts and pollen grain volumes. “Floral unit” is the scale at which pollen was collected from species, with capitulum measurements for members of Asteraceae and individual flower measurements for all other species. “Source species” were used when pollen production values were only available for morphologically similar species within the same genus and is left blank when the species itself was used.

| Species | Pollen  (μl/floral unit) | Source species | Literature source |
| --- | --- | --- | --- |
| *Acer spicatum* | 0.02 |  | (Biesboer 1975, Sullivan 1983) |
| *Achillea millefolium* | 1.13 |  | (Hicks et al. 2016) |
| *Actaea pachypoda* | 0.52 |  | (Pellmyr 1985); <http://www.discoverlife.org> |
| *Capsella bursa-pastoris* | 0.001 |  | (Hicks et al. 2016) |
| *Centaurea* sp*.* | 6.40 | *C. nigra* | (Hicks et al. 2016) |
| *Cerastium fontanum* | 0.04 |  | (Hicks et al. 2016) |
| *Cucumis melo* | 0.13 |  | (Perveen and Qaiser 2008, Kouonon et al. 2009) |
| *Cucurbita pepo* (zucchini) | 13.1 |  | (Nepi and Pacini 1993, Vidal et al. 2006) |
| *Cucurbita sp.* (squash) | 34.8 |  | (Vidal et al. 2006) |
| *Daucus carota* | 0.02 |  | (Hicks et al. 2016) |
| *Echium vulgare* | 0.15 |  | (Hicks et al. 2016) |
| *Erythronium americanum* | 16.3 |  | (Harder et al. 1985, Kosenko 1999) |
| *Fagopyrum esculentum* | 0.06 |  | (Cawoy et al. 2006) |
| *Fragaria virginiana* | 1.42 |  | (Hebda et al. 1988, Ashman and Hitchens 2000) |
| *Galium mollugo* | 0.007 | *G. verum,*  *G. album* | (Hicks et al. 2016) |
| *Galium palustre* | 0.007 | *G. verum,*  *G. album* | (Hicks et al. 2016) |
| *Glechoma hederacea* | 0.06 |  | (Hutchings and Price 1999, Moon et al. 2008) |
| *Leucanthemum vulgare* | 15.9 |  | (Hicks et al. 2016) |
| *Linaria vulgaris* | 0.44 |  | (Olsson 1974, Arnold 1982) |
| *Lotus corniculatus* | 0.15 |  | (Hicks et al. 2016) |
| *Malva neglecta* | 0.58 |  | (Cruden 1977); <http://blogs.cornell.edu/pollengrains/> |
| *Medicago sativa* | 0.16 |  | (Müller et al. 2006) |
| *Oenothera biennis* | 8.00 |  | (Cruden and Jensen 1979, Hall et al. 1988) |
| *Plantago lanceolata* | 0.01 |  | (Hicks et al. 2016) |
| *Prunella vulgaris* | 0.03 |  | (Hicks et al. 2016) |
| *Ranunculus acris* | 1.40 |  | (Hicks et al. 2016) |
| *Silene vulgaris* | 0.36 | *S. dioica,*  *S. latifolia* | (Hicks et al. 2016) |
| *Sonchus arvensis* | 0.94 | *S. asper* | (Hicks et al. 2016) |
| *Trifolium aureum* | 2.82 |  | (Hicks et al. 2016) |
| *Trifolium repens* | 0.02 |  | (Hicks et al. 2016) |
| *Trillium erectum* | 0.03 |  | (Hicks et al. 2016) |
| *Veronica* sp. | 0.03 | *V. persica* | (Hicks et al. 2016) |
| *Vicia cracca* | 0.04 |  | (Hicks et al. 2016) |

Table S6 Pearson correlation coefficients for fixed effects in all models run when unknown areas in the landscape were assigned either the median (‘Median’) or the minimum (‘Minimum’) floral resource volume across all known floral resource-providing land types. ‘FR’ = floral resource volume, ‘T1’ = late spring, ‘T2’ = early summer, ‘T3’ = mid-summer, and ‘T4’ = late summer. Coefficient values range from r = –0.38 to 0.28. Blank cells represent fixed effect pairs that did not occur within the same model.

|  | | TransectFR (T1–4) | TransectFR (T2–3) | TransectFR (T3–4) | ChangeInFR at 250m | ChangeInFR at 500m | ChangeInFR at 750m | non–*Andrena* visits (T1–4) | non–*Bombus* visits (T1–4) | non–*Halictus* visits (T1–4) | non–*Lasioglossum* visits (T1–4) | non–*Megachile* visits (T2–3) | non–*Peponapis* visits (T3–4) |
| --- | --- | --- | --- | --- | --- | --- | --- | --- | --- | --- | --- | --- | --- |
|  | TransectFR |  |  |  |  |  |  | 0.13 | 0.12 | 0.13 | 0.16 | 0.01 | –0.38 |
| Median | PresentFR at 250m | 0.20 | 0.16 | 0.24 |  |  |  | 0.16 | 0.16 | 0.13 | 0.15 | –0.01 | –0.25 |
|  | PresentFR at 500m | 0.18 | 0.14 | 0.12 |  |  |  | 0.05 | 0.08 | 0.03 | 0.05 | –0.12 | –0.24 |
|  | PresentFR at 750m | 0.17 | 0.11 | 0.12 |  |  |  | –0.01 | 0.02 | –0.01 | 0.01 | –0.16 | –0.25 |
|  | ChangeInFR at 250m | 0.04 | 0.17 |  |  |  |  | –0.06 |  | –0.09 | –0.09 | 0.10 |  |
|  | ChangeInFR at 500m | 0.04 | 0.16 |  |  |  |  | –0.08 |  | –0.11 | –0.11 | –0.04 |  |
|  | ChangeInFR at 750m | 0.04 | 0.15 |  |  |  |  | –0.11 |  | –0.14 | –0.14 | –0.08 |  |
|  | FR in T1 at 250m | 0.22 |  |  | –0.29 | –0.22 | –0.16 | 0.11 |  | 0.11 | 0.13 |  |  |
|  | FR in T1 at 500m | 0.17 |  |  | –0.19 | –0.16 | –0.10 | –0.01 |  | 0.00 | 0.01 |  |  |
|  | FR in T1 at 750m | 0.13 |  |  | –0.12 | –0.12 | –0.06 | –0.08 |  | –0.05 | –0.05 |  |  |
|  | FR in T2 at 250m |  | 0.11 |  | 0.03 | 0.10 | 0.04 |  | 0.12 |  |  | –0.09 |  |
|  | FR in T2 at 500m |  | 0.09 |  | 0.17 | 0.20 | 0.08 |  | 0.04 |  |  | –0.16 |  |
|  | FR in T2 at 750m |  | 0.07 |  | 0.19 | 0.23 | 0.13 |  | –0.01 |  |  | –0.17 |  |
|  | FR in T3 at 250m |  |  | 0.24 |  |  |  |  |  |  |  |  | –0.26 |
|  | FR in T3 at 500m |  |  | 0.12 |  |  |  |  |  |  |  |  | –0.24 |
|  | FR in T3 at 750m |  |  | 0.11 |  |  |  |  |  |  |  |  | –0.25 |
|  | CumulativeFR at 250m | 0.15 |  |  |  |  |  |  | 0.12 | 0.16 | 0.18 |  |  |
|  | CumulativeFR at 500m | 0.11 |  |  |  |  |  |  | 0.04 | 0.08 | 0.11 |  |  |
|  | CumulativeFR at 750m | 0.08 |  |  |  |  |  |  | –0.01 | 0.07 | 0.08 |  |  |
| Minimum | PresentFR at 250m | 0.21 | 0.16 | 0.28 |  |  |  | 0.17 | 0.16 | 0.14 | 0.16 | 0.01 | –0.28 |
|  | PresentFR at 500m | 0.18 | 0.14 | 0.15 |  |  |  | 0.05 | 0.08 | 0.04 | 0.06 | –0.11 | –0.24 |
|  | PresentFR at 750m | 0.17 | 0.11 | 0.14 |  |  |  | –0.01 | 0.02 | 0.00 | 0.01 | –0.16 | –0.24 |
|  | ChangeInFR at 250m | 0.02 | 0.19 |  |  |  |  | –0.02 |  | –0.05 | –0.05 | 0.16 |  |
|  | ChangeInFR at 500m | 0.03 | 0.18 |  |  |  |  | –0.05 |  | –0.07 | –0.07 | 0.06 |  |
|  | ChangeInFR at 750m | 0.04 | 0.14 |  |  |  |  | –0.09 |  | –0.11 | –0.11 | –0.06 |  |
|  | FR in T1 at 250m | 0.22 |  |  | –0.37 | –0.29 | –0.19 | 0.11 |  | 0.10 | 0.12 |  |  |
|  | FR in T1 at 500m | 0.17 |  |  | –0.30 | –0.25 | –0.16 | –0.01 |  | 0.00 | 0.01 |  |  |
|  | FR in T1 at 750m | 0.13 |  |  | –0.22 | –0.20 | –0.14 | –0.07 |  | –0.05 | –0.05 |  |  |
|  | FR in T2 at 250m |  | 0.11 |  | 0.09 | 0.13 | 0.05 |  |  |  |  | –0.07 |  |
|  | FR in T2 at 500m |  | 0.10 |  | 0.17 | 0.18 | 0.14 |  |  |  |  | –0.15 |  |
|  | FR in T2 at 750m |  | 0.07 |  | 0.18 | 0.21 | 0.14 |  |  |  |  | –0.16 |  |
|  | FR in T3 at 250m |  |  | 0.28 |  |  |  |  |  |  |  |  | –0.28 |
|  | FR in T3 at 500m |  |  | 0.14 |  |  |  |  |  |  |  |  | –0.24 |
|  | FR in T3 at 750m |  |  | 0.14 |  |  |  |  |  |  |  |  | –0.24 |
|  | CumulativeFR at 250m | 0.05 |  |  |  |  |  |  | 0.04 | 0.13 | 0.15 |  |  |
|  | CumulativeFR at 500m | 0.01 |  |  |  |  |  |  | 0.01 | 0.09 | 0.11 |  |  |
|  | CumulativeFR at 750m | 0.00 |  |  |  |  |  |  | –0.01 | 0.09 | 0.11 |  |  |

Table S7 Format of fixed effect terms in all models run, presented in R syntax for the following hypotheses: ‘H0’ = bee visits are not limited by floral resources; ‘H1’ = bee visits are only influenced by the present abundance of local floral resources; ‘H2’ = bee visits are influenced by the present abundance of landscape floral resources; ‘H3a’ = both the abundance of floral resources in the landscape when foraging begins and any decreases in the abundance of floral resources later in the season will best predict bee visits; and ‘H3b’ = the cumulative abundance of landscape floral resources from when foraging begins will best predict bee visits. Models included a log offset to account for varying lengths of observation time based on transect sizes, and the crossed random effects of time period and site.

| Hypothesis | Models |
| --- | --- |
| H0 | Visits ~ AllOtherVisits |
|  | Visits ~ 1 |
| H1 | Visits ~ TransectFR + AllOtherVisits |
|  | Visits ~ TransectFR |
| H2 | Visits ~ PresentFR + TransectFR + AllOtherVisits |
|  | Visits ~ PresentFR + TransectFR |
|  | Visits ~ PresentFR + AllOtherVisits |
|  | Visits ~ PresentFR |
| H3a | Visits ~ FirstTimePeriodFR + ChangeInFR + TransectFR + AllOtherVisits |
|  | Visits ~ FirstTimePeriodFR + ChangeInFR + TransectFR |
|  | Visits ~ FirstTimePeriodFR + ChangeInFR + AllOtherVisits |
|  | Visits ~ FirstTimePeriodFR + ChangeInFR |
| H3b | Visits ~ CumulativeFR + TransectFR + AllOtherVisits |
|  | Visits ~ CumulativeFR + TransectFR |
|  | Visits ~ CumulativeFR + AllOtherVisits |
|  | Visits ~ CumulativeFR |
